# Reference values for handgrip strength in Europe: analysis of individual participant data from 27 countries

**DOI:** 10.1007/s11357-025-01919-9

**Published:** 2025-10-01

**Authors:** Jozo Grgic, Vanessa Kristina Wazny, Andrea B. Maier, Brad J. Schoenfeld, Zeljko Pedisic

**Affiliations:** 1 NUS Academy for Healthy Longevity, Yong Loo Lin School of Medicine, National University of Singapore (NUS), Singapore, 117456 Singapore; 2 Department of Human Movement Sciences, @AgeAmsterdam, Faculty of Behavioural and Movement Sciences, Vrije Universiteit Amsterdam, Amsterdam Movement Sciences, Van Der Boechorststraat 7, 1081 BT Amsterdam, The Netherlands; 3 Healthy Longevity Translational Research Programme, Yong Loo Lin School of Medicine, National University of Singapore, Singapore, 117597 Singapore; 4 Department of Exercise Science and Recreation, Applied Muscle Development Lab, CUNY Lehman College, Bronx, NY USA; 5 School of Public Health, Li Ka Shing Faculty of Medicine, The University of Hong Kong, Hong Kong, China

**Keywords:** Normative data, SHARE, Grip, Surveillance, Centiles, Norms

## Abstract

We aimed to determine reference values for handgrip strength in Europe. Data from the Survey of Health, Ageing and Retirement in Europe were used, involving adults aged 50 + years. Reference values were expressed as weighted percentiles for absolute (kg) and relative (kg/m^2^) handgrip strength. For women, the highest absolute handgrip strength was observed among the 50–54-year-olds (standing: 5th percentile [P5] = 19 kg; 50th percentile [P50] = 29 kg; 95th percentile [P95] = 39 kg, sitting: P5 = 20 kg; P50 = 29 kg; P95 = 40 kg) and the lowest among the ≥ 90-year-olds (standing: P5 = 9 kg; P50 = 17 kg; P95 = 26 kg, sitting: P5 = 6 kg; P50 = 14 kg; P95 = 24 kg). For men, the highest absolute handgrip strength was observed among the 50–54-year-olds (standing: P5 = 30 kg; P50 = 46 kg; P95 = 62 kg, sitting: P5 = 29 kg; P50 = 46 kg; P95 = 60 kg) and the lowest among the ≥ 90-year-olds (standing: P5 = 12 kg; P50 = 27 kg; P95 = 39 kg, sitting: P5 = 10 kg; P50 = 25 kg; P95 = 37 kg). Compared with women, men had higher handgrip strength. Handgrip strength was generally higher in Northern and Western Europe compared with Southern and Central/Eastern Europe, and in the standing position compared with the sitting position. Similar patterns were found for relative handgrip strength. The reference values determined in this study can facilitate benchmarking for clinical, population health, and research purposes.

**Supplementary Information:**

The online version contains supplementary material available at 10.1007/s11357-025-01919-9.

## Introduction

Handgrip strength is widely recognised as a biomarker of overall health, particularly in older adults [1]. Low handgrip strength has been consistently associated with adverse outcomes, including functional decline, frailty, falls, prolonged hospitalisation, disability, cognitive impairment, chronic disease, and higher all-cause mortality risk [2–8]. These associations have been found even in initially healthy individuals, underscoring the value of handgrip test as a proxy for biological resilience and ageing-related vulnerability [9, 10]. Given that this test is non-invasive, easy to administer, and highly reliable across different populations (intraclass correlation coefficients ≥ 0.90), it has been widely used in both clinical practice and research [9–11].

Reference handgrip strength values can serve as a benchmark for identifying individuals at an increased risk of adverse health outcomes. However, previous European studies on reference values of the handgrip test were generally based on data from a single country or specific population, which limits their generalisability [12–14]. For example, one recent study provided reference data for community-dwelling adults aged 65–75 years in Germany [12], while another provided reference values based on a regional sample from South-East Poland [13]. Even a large study based on pooled analysis of 12 cohort studies was focused exclusively on one country, namely, the UK [14]. In a recent systematic review, reference values for handgrip strength were determined by combining data from 69 countries and regions [15]. However, no data were provided specifically for Europe. Given that handgrip strength may vary substantially across different geographic regions and populations [12–17], there is a need to determine cross-national, standardised reference values for handgrip strength in Europe.

A previous study examined handgrip strength in 11 European countries, using data from the first wave of the Survey of Health, Ageing and Retirement in Europe (SHARE) study [18]. The results revealed lower handgrip strength in Southern Europe, suggesting a north–south gradient in muscle strength. However, the study reported only mean values rather than reference percentiles, limiting its utility for clinical benchmarking. Additionally, there was a lack of data from Central and Eastern Europe in the first wave of SHARE, which limits the generalisability of the findings. It should also be noted that the study was based on data from the first wave of SHARE, conducted in 2004. A subsequent analysis of data from SHARE waves 1–5 explored time trends in handgrip strength in Germany, Spain, and Sweden from 2004 to 2013 [19]. The study found an upward trend in handgrip strength among 80 + year-olds, while no change or downward trend were found among younger older adults [19]. These findings suggest that reference values for handgrip strength need to be periodically updated, because historical data may no longer accurately reflect the functional capacity of today’s populations.

The aim of this study was to determine up-to-date reference values for handgrip strength among adults aged 50 + years, based on national-representative data from 27 European countries.

## Material and methods

### Study design and sample

We used data from the SHARE cohort, a large-scale, longitudinal study involving non-institutionalized individuals aged 50 + years and their spouses in 28 European countries and Israel. Since its launch in 2004, SHARE has been conducted nine times [20]. For our analysis, we used data collected during the 2021–2022 period, corresponding to the ninth wave of the survey. This wave marked the most recent instance in which handgrip strength measurements were taken. We used data collected among 27 countries, including: Austria, Belgium, Bulgaria, Croatia, Cyprus, Czech Republic, Denmark, Estonia, Finland, France, Germany, Greece, Hungary, Italy, Latvia, Lithuania, Luxembourg, Malta, Netherlands, Poland, Portugal, Romania, Slovakia, Slovenia, Spain, Sweden, and Switzerland. The analyses included data from 25,708 men and 32,801 women. All participants signed informed consent forms before participating in the study, and ethical approval was obtained from the Ethics Council of the Max Planck Society, as well as from national ethics bodies in each participating nation. More detailed information on the study design and procedures can be found in previous publications [20–23].

### Handgrip strength

Grip strength was assessed using a mechanical handgrip dynamometer (Smedley Dynamometer, TTM, Tokyo). The participants performed the test with their elbows bent at a 90-degree angle (i.e., in the handshake position) and their wrists held in neutral position. While the test was designed to be performed standing, participants were also allowed to perform it seated, based on their preference. Two measurements were taken for each arm. In this study, we used data on the maximum strength of the dominant arm only, in line with recommendations [24]. Upon completion of the test, the assessors asked individuals to rate the level of effort they exerted during the task. Participants indicated if they exerted maximum effort or were unable to do so due to health-related or other, unspecified factors. For the purpose of this analysis, only data from those who reported exerting their maximum effort were included. Handgrip strength was expressed in absolute (kg) and relative (kg divided by body height in m^2^) units. Body height was used in the calculation of relative handgrip strength, because it has been identified through allometric analyses as the most appropriate body size dimension for this purpose [25–28].

### Sociodemographic, health, lifestyle, and morphological characteristics

The following sociodemographic data were used: sex; age (categorized as 50–54, 55–59, 60–64, 65–69, 70–74, 75–79, 80–84, 85–89, and 90 + years of age); employment status (categorized as “employed or self-employed”, “unemployed”, “retired”, “not working due to permanent sickness or disability”, “homemaker”, and “other”); and residence (categorized as “home” and “nursing home”). The following health-related variables were used: self-rated health status (categorized as “excellent”, “very good”, “good”, “fair”, and “poor”); functional status, estimated via the self-reported number of difficulties encountered in performing five basic activities of daily living and scored on a scale from 0 to 5; self-reported mobility limitations scored from 0 to 7; and body mass index (BMI), categorized as < 18.5, 18.5–24.9, 25.0–29.9, 30.0–34.9, 35.0–39.9, and > 40.0 kg/m^2^. The following lifestyle variables were used: smoking status (categorized as “current smoker” and “non-smoker”); self-reported frequency of participation in moderate physical activity; and self-reported frequency of participation in vigorous physical activity, both categorized as “ > 1 time per week”, “1 time per week”, “1–3 times per month”, and “hardly ever or never”. We also used data on participants’ body height, expressed in metres.

### Statistical analysis

We calculated absolute frequencies and weighted percentages for all sociodemographic, health, and lifestyle categories. Height was reported in meters as mean ± standard deviation. The participants were grouped into nine age categories, and reference values in handgrip strengths were calculated for each of the groups as the 5th, 10th, 20th, 30th, 40th, 50th, 60th, 70th, 80th, 90th, and the 95th weighted percentile. Analyses were performed using data on absolute and relative handgrip strength in the standing and sitting positions separately, as well as using combined data for both positions. In the main analysis, we pooled data from all countries. Subgroup analyses were conducted to calculate reference values according to regions of Europe, including Central and Eastern Europe (Bulgaria, Croatia, Czech Republic, Hungary, Poland, Romania, Slovakia, and Slovenia), Northern Europe (Denmark, Estonia, Finland, Latvia, Lithuania, and Sweden), Southern Europe (Cyprus, Greece, Italy, Malta, Portugal, and Spain), and Western Europe (Austria, Belgium, France, Germany, Luxembourg, Netherlands, and Switzerland), using the EuroVoc classification [29]. All analyses were conducted separately for men and women. To improve the generalizability of the results, the sample weights from the SHARE dataset were applied. All analyses were conducted using IBM SPSS Statistics, version 30 (SPSS Inc., an IBM Company, Chicago, IL, USA).

## Results

### Participant characteristics

Most participants were older adults, in both male (65.9%) and female (62.9%) samples (Table 1). According the employment status, the most represented group were retirees (men: 49.3%, women: 46.7%), followed by those who were employed or self-employed (men: 40.5%, women: 32.0%), homemakers among women (13.1%), the unemployed among men (4.4%), and other groups (men: 5.1%, women: 7.4%). At the time of the study, nearly all participants (99.3% in both sexes) lived at home, while the remaining participants lived in nursing homes. The self-reported health status was good, very good, or excellent for 69.2% men and 66.9% women in the sample. A vast majority of participants (men: 92.9%, women: 92.2%) reported that they do not require assistance with activities of daily living, while most participants (men: 66.6%, women: 53.3%) stated that they do not have any mobility limitations. According to BMI, most participants were classified as “normal” weight (men: 30.5%, women: 39.9%) or overweight (men: 46.1%, women: 34.7%). The participants in the sample were predominantly non-smokers (men: 79.0%, women: 85.4%). While most participants (men: 67.1%, women: 63.4%) reported engaging in moderate-intensity physical activity more than once per week, such regular participation in vigorous physical activity was less prevalent (men: 40.5%, women: 30.7%). The participants’ height, for all countries combined as well as for specific regions of Europe, is reported in Table 2.

**Table 1:** Participant characteristics

Characteristic	Frequency (weighted percentage)	
	Women (*n* = 32,801)	Men (*n* = 25,708)
*Age (years)*		
50–54	1,936 (54.9)	1,078 (45.1)
55–59	4,328 (48.2)	3,072 (51.8)
60–64	5,638 (50.2)	4,436 (49.8)
65–69	6,156 (51.8)	5,116 (48.2)
70–74	5,780 (52.9)	4,929 (47.1)
75–79	4,283 (54.1)	3,525 (45.9)
80–84	2,871 (58.2)	2,215 (41.8)
85–89	1,312 (58.6)	1,000 (41.4)
90 +	497 (63.6)	337 (36.4)
*Employment status*		
Employed or self-employed	7,067 (32.0)	6,181 (40.5)
Unemployed	585 (2.5)	542 (4.4)
Retired	20,261 (46.7)	17,893 (49.3)
Not working due to permanent sickness or disability	676 (3.0)	625 (3.4)
Homemaker	3,428 (13.1)	201 (0.8)
Other	531 (1.9)	98 (0.9)
*Residence*		
Home	32,559 (99.3)	25,549 (99.3)
Nursing home	192 (0.4)	116 (0.3)
*Self-reported health*		
Excellent	1,872 (6.1)	1,600 (7.0)
Very good	5,618 (18.7)	4,492 (20.1)
Good	13,453 (42.1)	10,753 (42.1)
Fair	9,356 (26.2)	7,005 (24.3)
Poor	2,493 (6.9)	1,847 (6.5)
*Activities of daily living score*^a^		
0	30,174 (92.2)	23,771 (92.9)
1	1,548 (4.5)	1,258 (4.4)
2	577 (1.6)	403 (1.5)
3	256 (0.8)	187 (0.8)
4	150 (0.4)	90 (0.3)
5	80 (0.3)	51 (0.2)
*Mobility score*^b^		
0	16,200 (53.3)	15,661 (66.6)
1	5,305 (15.4)	3,983 (14.9)
2	4,093 (11.5)	2,528 (8.0)
3	2,995 (8.2)	1,619 (4.9)
4	2,049 (5.8)	955 (2.7)
5	1264 (3.2)	556 (1.7)
6	649 (1.9)	256 (0.7)
7	240 (0.8)	138 (0.5)
*Body mass index (kg/m*^*2*^*)*		
< 18.5	513 (2.1)	109 (0.5)
18.5–24.9	11,627 (39.9)	7,298 (30.5)
25.0–29.9	11,867 (34.7)	11,851 (46.1)
30.0–34.9	5,617 (14.2)	4,677 (16.6)
35.0–39.9	1,705 (4.3)	1,054 (3.4)
> 40.0	559 (1.5)	295 (1.0)
*Smoking status*		
Non-smoker	28,535 (85.4)	20,969 (79.0)
Current smoker	4,260 (14.6)	4,724 (21.0)
*Moderate physical activity*		
> 1 time per week	22,222 (63.4)	17,943 (67.1)
1 time per week	4,464 (15.2)	3,492 (15.0)
1–3 times per month	2,245 (7.9)	1,793 (8.0)
Hardly ever or never	3,862 (13.5)	2,477 (9.9)
*Vigorous physical activity*		
> 1 time per week	9,718 (30.7)	9,787 (40.5)
1 time per week	4,742 (13.4)	3,684 (13.2)
1–3 times per month	3,751 (9.9)	2,947 (10.3)
Hardly ever or never	14,576 (46.0)	9,278 (36.0)

^a^Higher score indicates lower functional independence

^b^Higher score indicates lower mobility

**Table 2:** Arithmetic means ± standard deviations of participant body height in meters

Age (years)	All countries	Central and Eastern Europe	Northern Europe	Southern Europe	Western Europe
*Women*					
50–54	1.66 ± 0.06	1.66 ± 0.06	1.66 ± 0.06	1.62 ± 0.06	1.66 ± 0.06
55–59	1.65 ± 0.06	1.65 ± 0.06	1.66 ± 0.06	1.62 ± 0.06	1.66 ± 0.06
60–64	1.64 ± 0.06	1.64 ± 0.06	1.66 ± 0.06	1.62 ± 0.06	1.65 ± 0.06
65–69	1.64 ± 0.06	1.64 ± 0.06	1.65 ± 0.06	1.61 ± 0.06	1.64 ± 0.06
70–74	1.63 ± 0.06	1.63 ± 0.06	1.64 ± 0.06	1.60 ± 0.06	1.64 ± 0.06
75–79	1.62 ± 0.06	1.62 ± 0.06	1.64 ± 0.06	1.60 ± 0.06	1.63 ± 0.06
80–84	1.62 ± 0.07	1.62 ± 0.07	1.62 ± 0.06	1.59 ± 0.06	1.63 ± 0.06
85–89	1.61 ± 0.07	1.61 ± 0.06	1.61 ± 0.07	1.59 ± 0.07	1.61 ± 0.06
90 +	1.60 ± 0.06	1.61 ± 0.06	1.61 ± 0.06	1.58 ± 0.07	1.61 ± 0.06
*Men*					
50–54	1.79 ± 0.07	1.78 ± 0.07	1.80 ± 0.07	1.74 ± 0.07	1.79 ± 0.07
55–59	1.77 ± 0.07	1.77 ± 0.07	1.80 ± 0.07	1.74 ± 0.07	1.78 ± 0.07
60–64	1.77 ± 0.07	1.76 ± 0.07	1.79 ± 0.07	1.73 ± 0.08	1.78 ± 0.07
65–69	1.76 ± 0.07	1.75 ± 0.07	1.78 ± 0.06	1.72 ± 0.07	1.78 ± 0.07
70–74	1.75 ± 0.07	1.75 ± 0.07	1.77 ± 0.07	1.71 ± 0.07	1.76 ± 0.07
75–79	1.74 ± 0.07	1.74 ± 0.07	1.77 ± 0.06	1.70 ± 0.07	1.76 ± 0.07
80–84	1.74 ± 0.07	1.73 ± 0.07	1.76 ± 0.06	1.70 ± 0.06	1.74 ± 0.07
85–89	1.73 ± 0.07	1.73 ± 0.06	1.76 ± 0.06	1.69 ± 0.08	1.74 ± 0.07
90 +	1.72 ± 0.07	1.73 ± 0.06	1.74 ± 0.06	1.67 ± 0.07	1.72 ± 0.07

### Pan-European reference values for handgrip strength in the standing position

#### Absolute handgrip strength

For women, the highest handgrip strength was observed among the 50–54-year-olds (5th percentile = 19 kg; 50th percentile = 29 kg; 95th percentile = 39 kg; Table 3) and the lowest among the ≥ 90-year-olds (5th percentile = 9 kg; 50th percentile = 17 kg; 95th percentile = 26 kg). For men, the highest handgrip strength was observed among the 50–54-year-olds (5th percentile = 30 kg; 50th percentile = 46 kg; 95th percentile = 62 kg; Table 4) and the lowest among the ≥ 90-year-olds (5th percentile = 12 kg; 50th percentile = 27 kg; 95th percentile = 39 kg). Compared with women, men had higher handgrip strength in all 99 percentile-by-age comparisons, with the range of differences between the respective percentiles (R_d_) from 3 to 23 kg.

**Table 3:** Reference values for absolute handgrip strength in the standing position for women

Age (years)	*n*	Weighted percentile (kg)										
		5th	10th	20th	30th	40th	50th	60th	70th	80th	90th	95th
Europe (pooled *n* = 24,594)												
50–54	1,527	19	21	24	26	28	29	31	32	34	37	39
55–59	3,409	19	21	24	25	27	28	30	32	33	36	38
60–64	4,509	19	21	24	25	26	28	29	31	32	35	37
65–69	4,831	17	19	22	24	25	26	28	29	31	34	36
70–74	4,389	15	17	20	22	23	25	26	28	29	32	34
75–79	3,077	13	15	18	20	21	23	24	25	27	30	32
80–84	1,882	12	14	17	18	20	21	22	24	25	28	30
85–89	747	10	12	14	16	17	19	20	22	23	25	27
90 +	223	9	11	13	15	16	17	18	19	20	23	26
Central and Eastern Europe (pooled *n* = 7,980)												
50–54	543	20	23	25	27	28	29	30	32	33	36	39
55–59	1,241	19	21	24	26	27	28	30	32	33	36	38
60–64	1,557	18	20	23	25	27	28	29	31	33	35	37
65–69	1,707	16	19	22	23	25	26	27	29	30	34	35
70–74	1,422	15	17	20	22	23	25	26	28	30	32	35
75–79	862	12	15	17	19	21	22	24	25	27	30	33
80–84	464	10	13	15	17	19	20	22	23	24	28	30
85–89	154	11	13	15	16	17	19	20	22	23	25	25
90 +	30	11	11	11	12	13	15	15	18	20	24	25
Northern Europe (pooled *n* = 5,521)												
50–54	336	24	25	27	28	30	31	33	36	38	39	41
55–59	659	21	24	26	28	29	30	32	33	35	38	41
60–64	908	20	22	24	25	27	29	30	32	33	36	38
65–69	990	18	20	23	25	26	28	29	30	32	34	37
70–74	962	18	20	22	24	25	27	28	29	31	33	35
75–79	808	15	18	20	22	23	24	26	27	29	31	33
80–84	552	13	15	17	20	21	22	23	25	26	28	30
85–89	230	12	13	16	18	19	21	22	23	25	27	29
90 +	76	10	11	14	15	17	18	19	20	20	25	27
Southern Europe (pooled *n* = 3,897)												
50–54	198	16	20	22	26	26	28	29	32	33	36	37
55–59	546	18	21	22	23	25	26	28	30	32	36	38
60–64	731	17	21	23	24	25	27	28	30	31	33	36
65–69	744	15	18	20	22	24	25	26	28	30	32	34
70–74	729	13	16	18	20	21	23	24	25	28	30	33
75–79	525	11	14	16	19	20	22	23	25	26	28	31
80–84	279	10	14	15	18	19	20	21	23	25	28	30
85–89	116	6	10	12	13	15	16	18	20	22	25	27
90 +	29	4	9	14	15	16	18	18	18	19	20	20
Western Europe (pooled *n* = 7,196)												
50–54	450	19	22	25	27	28	30	31	32	35	37	39
55–59	963	20	23	25	27	28	29	30	32	34	36	39
60–64	1,313	20	21	24	25	27	28	30	31	33	35	37
65–69	1,390	18	20	23	24	25	27	28	30	31	34	36
70–74	1,276	17	19	21	23	24	25	27	28	30	32	33
75–79	882	15	17	19	20	22	23	25	26	27	29	31
80–84	587	13	15	17	19	20	22	23	24	25	28	29
85–89	247	10	14	15	17	18	20	21	22	23	26	27
90 +	88	11	12	13	14	16	17	18	20	23	25	26

The following classification of countries to European regions was used: Central and Eastern Europe (Bulgaria, Croatia, Czech Republic, Hungary, Poland, Romania, Slovakia, and Slovenia); Northern Europe (Denmark, Estonia, Finland, Latvia, Lithuania, and Sweden); Southern Europe (Cyprus, Greece, Italy, Malta, Portugal, and Spain); and Western Europe (Austria, Belgium, France, Germany, Luxembourg, Netherlands, and Switzerland)

**Table 4:** Reference values for absolute handgrip strength in the standing position for men

Age (years)	*n*	Weighted percentile (kg)										
		5th	10th	20th	30th	40th	50th	60th	70th	80th	90th	95th
Europe (pooled *n* = 19,620)												
50–54	852	30	35	39	41	44	46	48	51	54	58	62
55–59	2,474	30	34	37	40	42	45	47	50	52	56	59
60–64	3,502	27	31	36	40	42	44	46	48	51	55	57
65–69	3,976	27	31	35	37	40	42	44	46	49	52	55
70–74	3,826	25	28	32	35	37	39	41	44	46	49	52
75–79	2,626	22	25	29	31	34	36	38	40	43	46	48
80–84	1,574	20	22	27	30	31	33	35	38	40	43	45
85–89	622	17	20	23	25	28	29	31	33	36	40	43
90 +	168	12	17	21	23	25	27	29	30	33	36	39
Central and Eastern Europe (pooled *n* = 6,282)												
50–54	239	29	32	36	40	42	45	47	49	52	58	62
55–59	892	27	31	36	39	42	45	46	48	50	55	58
60–64	1,247	27	30	35	37	40	42	44	46	49	53	56
65–69	1,405	25	29	34	35	38	41	43	45	47	52	54
70–74	1,274	24	27	32	34	35	38	40	42	44	48	50
75–79	702	21	25	29	31	33	35	38	40	42	45	49
80–84	358	19	23	26	29	30	32	34	37	40	43	45
85–89	135	20	22	25	26	28	30	33	36	42	45	45
90 +	30	12	16	20	23	24	24	26	29	37	39	39
Northern Europe (pooled *n* = 4,181)												
50–54	225	35	39	44	48	50	52	53	56	58	63	64
55–59	543	35	39	41	44	45	48	50	53	55	58	60
60–64	731	31	36	40	42	45	47	49	51	54	58	61
65–69	758	28	33	37	40	42	44	46	48	51	54	58
70–74	762	29	32	36	38	40	42	44	46	48	52	54
75–79	606	26	29	33	35	37	39	41	43	45	48	51
80–84	380	24	27	30	32	34	35	38	40	42	44	48
85–89	136	21	24	27	28	30	32	34	36	37	41	45
90 +	40	15	17	24	26	27	30	31	32	35	40	40
Southern Europe (pooled *n* = 3,195)												
50–54	77	25	33	35	39	40	42	45	49	50	52	55
55–59	330	28	32	37	39	41	42	44	46	49	52	56
60–64	511	25	27	33	36	39	41	44	46	50	52	53
65–69	668	25	28	32	34	36	39	41	43	45	49	51
70–74	625	24	26	30	32	35	37	39	40	43	46	50
75–79	510	20	24	26	29	31	33	35	38	40	43	46
80–84	306	17	20	24	27	29	31	33	35	38	41	43
85–89	142	15	18	21	24	26	29	31	32	35	39	43
90 +	26	12	18	22	25	26	30	30	30	32	34	35
Western Europe (pooled *n* = 5,962)												
50–54	311	33	37	40	44	46	48	51	53	56	60	63
55–59	709	33	36	40	42	44	46	49	51	53	57	61
60–64	1,013	31	35	40	42	45	46	48	50	53	55	58
65–69	1,145	31	34	37	40	42	44	45	47	50	53	56
70–74	1,165	29	32	35	37	40	41	43	45	48	50	53
75–79	808	25	28	31	34	36	38	40	42	45	47	49
80–84	530	22	26	29	31	34	35	38	39	42	44	47
85–89	209	18	22	24	27	28	30	31	34	37	40	42
90 +	72	18	21	24	27	28	30	31	33	35	40	42

The following classification of countries to European regions was used: Central and Eastern Europe (Bulgaria, Croatia, Czech Republic, Hungary, Poland, Romania, Slovakia, and Slovenia); Northern Europe (Denmark, Estonia, Finland, Latvia, Lithuania, and Sweden); Southern Europe (Cyprus, Greece, Italy, Malta, Portugal, and Spain); and Western Europe (Austria, Belgium, France, Germany, Luxembourg, Netherlands, and Switzerland)

#### Relative handgrip strength

For women, the highest relative handgrip strength was observed among the 50–54-year-olds and 55–59-year-olds (5th percentile = 7.4 kg/m^2^; 50th percentile = 10.7 kg/m^2^; 95th percentile = 14.2 kg/m^2^; Table 5) and the lowest among the ≥ 90-year-olds (5th percentile = 4.0 kg/m^2^; 50th percentile = 6.8 kg/m^2^; 95th percentile = 9.8 kg/m^2^), with an exception of the 5th percentile which was lower among the 85–89-year-olds (3.8 kg/m^2^). For men, the highest relative handgrip strength was observed among the 50–54-year-olds (5th percentile = 9.3 kg/m^2^; 50th percentile = 15.1 kg/m^2^; 95th percentile = 19.0 kg/m^2^; Table 6), with an exception of the 5th percentile which was higher among the 55–59-year-olds (9.7 kg/m^2^). The lowest relative handgrip strength among men was observed for the ≥ 90-year-olds (5th percentile = 5.1 kg/m^2^; 50th percentile = 9.7 kg/m^2^; 95th percentile = 14.0 kg/m^2^), with an exception of the 60th percentile which was lower among the 85–89-year-olds (10.4 kg/m^2^). Compared with women, men had higher relative handgrip strength in all 99 percentile-by-age comparisons (R_d_: 1.1 to 4.9 kg/m^2^).

**Table 5:** Reference values for relative handgrip strength in the standing position for women

Age (years)	*n*	Weighted percentile (kg/m^2^)										
		5th	10th	20th	30th	40th	50th	60th	70th	80th	90th	95th
Europe (pooled *n* = 24,443)												
50–54	1,521	7.2	8.0	9.0	9.6	10.2	10.7	11.3	11.8	12.6	13.6	14.1
55–59	3,395	7.4	8.1	8.8	9.4	9.9	10.4	11.1	11.6	12.3	13.3	14.2
60–64	4,488	7.0	7.9	8.8	9.3	9.9	10.3	10.9	11.4	12.0	12.9	13.7
65–69	4,808	6.4	7.3	8.3	8.9	9.4	9.8	10.4	10.9	11.6	12.5	13.2
70–74	4,371	5.8	6.7	7.6	8.3	8.8	9.3	9.9	10.4	11.1	12.0	12.7
75–79	3,055	5.1	5.9	7.0	7.6	8.1	8.6	9.2	9.8	10.4	11.2	12.0
80–84	1,858	4.8	5.5	6.3	7.0	7.6	8.1	8.6	9.0	9.6	10.4	11.2
85–89	733	3.8	4.7	5.6	6.2	6.9	7.4	8.0	8.7	9.0	9.9	10.8
90 +	214	4.0	4.3	5.0	5.6	6.5	6.8	7.1	7.3	8.1	9.6	9.8
Central and Eastern Europe (pooled *n* = 7,940)												
50–54	541	7.8	8.3	9.2	9.9	10.2	10.7	11.2	11.7	12.3	13.3	14.5
55–59	1,236	7.2	7.8	8.9	9.6	10.2	10.7	11.2	11.8	12.5	13.3	14.5
60–64	1,549	6.8	7.7	8.8	9.4	10.0	10.4	10.9	11.4	12.2	13.1	13.8
65–69	1,702	6.0	7.1	8.1	8.8	9.3	9.8	10.3	10.8	11.6	12.5	13.4
70–74	1,417	5.6	6.5	7.6	8.4	9.0	9.5	10.0	10.6	11.2	12.3	13.0
75–79	856	4.7	5.5	6.6	7.4	7.9	8.5	9.2	9.7	10.5	11.7	12.6
80–84	458	3.9	4.9	5.9	6.7	7.3	8.0	8.4	9.0	9.4	10.7	11.7
85–89	151	4.6	5.1	5.7	6.2	6.7	7.4	8.0	8.4	8.8	9.5	10.4
90 +	30	4.2	4.3	4.4	4.6	5.5	5.5	6.7	7.0	7.9	9.1	9.2
Northern Europe (pooled *n* = 5,507)												
50–54	336	8.8	9.2	9.9	10.5	11.0	11.5	12.1	12.6	13.3	13.9	14.2
55–59	658	7.4	8.2	9.5	10.2	10.6	10.9	11.4	11.8	12.4	13.5	14.3
60–64	907	7.2	8.1	9.0	9.4	9.9	10.5	11.0	11.7	12.2	12.9	13.8
65–69	989	6.8	7.7	8.5	9.1	9.7	10.0	10.5	11.1	11.6	12.5	13.5
70–74	959	6.5	7.4	8.3	8.8	9.3	9.8	10.3	10.7	11.2	12.0	12.8
75–79	805	5.9	6.7	7.4	8.0	8.4	9.0	9.5	10.0	10.7	11.5	12.1
80–84	551	5.2	5.9	6.6	7.3	7.9	8.3	8.7	9.2	9.8	10.7	11.4
85–89	229	5.0	5.5	6.4	7.1	7.4	8.2	8.6	8.9	9.3	9.9	11.0
90 +	73	4.2	4.4	5.4	6.1	6.6	7.0	7.5	7.8	8.4	9.4	10.9
Southern Europe (pooled *n* = 3,836)												
50–54	195	6.2	8.0	8.6	9.2	9.8	10.3	11.1	11.6	12.5	13.7	13.9
55–59	543	7.2	7.8	8.3	8.8	9.3	9.9	10.6	11.3	12.3	13.6	14.7
60–64	722	6.7	8.0	8.8	9.3	9.7	10.2	10.7	11.4	12.0	12.8	13.7
65–69	732	6.4	7.3	7.9	8.6	9.0	9.6	10.0	10.5	11.4	12.1	13.2
70–74	723	5.1	6.2	7.0	7.8	8.2	8.7	9.1	9.9	10.4	11.9	12.7
75–79	518	4.6	5.3	6.2	7.3	7.8	8.4	9.0	9.6	10.2	11.0	11.8
80–84	268	4.3	5.3	6.2	6.7	7.4	7.8	8.1	9.0	9.8	10.6	11.2
85–89	108	2.5	3.8	4.4	5.2	5.8	6.4	7.1	7.7	8.9	10.3	11.6
90 +	27	1.5	4.0	5.0	5.9	6.5	6.7	7.0	7.0	7.3	8.0	8.0
Western Europe (pooled *n* = 7,160)												
50–54	449	7.1	7.8	8.9	9.6	10.2	10.8	11.4	12.0	12.8	13.7	14.1
55–59	958	7.6	8.3	9.2	9.7	10.0	10.5	11.2	11.6	12.2	13.1	13.8
60–64	1,310	7.1	7.8	8.8	9.4	9.9	10.5	11.0	11.4	12.0	12.9	13.8
65–69	1,385	6.8	7.5	8.6	9.0	9.5	10.0	10.5	11.0	11.6	12.5	13.1
70–74	1,272	6.4	7.2	8.0	8.6	9.0	9.5	10.1	10.6	11.1	11.9	12.5
75–79	876	5.5	6.5	7.3	7.9	8.3	8.8	9.2	9.8	10.4	11.2	11.9
80–84	581	4.9	5.8	6.5	7.1	7.7	8.3	8.7	9.0	9.6	10.3	11.1
85–89	245	3.9	5.1	5.9	6.6	7.3	7.7	8.1	8.9	9.3	10.0	10.5
90 +	84	4.2	4.4	5.2	5.6	6.8	7.1	7.2	8.1	9.5	9.7	11.0

The following classification of countries to European regions was used: Central and Eastern Europe (Bulgaria, Croatia, Czech Republic, Hungary, Poland, Romania, Slovakia, and Slovenia); Northern Europe (Denmark, Estonia, Finland, Latvia, Lithuania, and Sweden); Southern Europe (Cyprus, Greece, Italy, Malta, Portugal, and Spain); and Western Europe (Austria, Belgium, France, Germany, Luxembourg, Netherlands, and Switzerland)

**Table 6:** Reference values for relative handgrip strength in the standing position for men

Age (years)	*n*	Weighted percentile (kg/m^2^)										
		5th	10th	20th	30th	40th	50th	60th	70th	80th	90th	95th
Europe (pooled *n* = 19,547)												
50–54	850	9.3	11.3	12.5	13.3	14.0	15.1	15.5	16.3	17.2	18.3	19.0
55–59	2,467	9.7	10.7	12.1	13.0	13.7	14.4	14.9	15.6	16.7	17.7	18.7
60–64	3,492	8.9	9.9	11.8	12.5	13.4	14.2	14.9	15.6	16.3	17.5	18.3
65–69	3,962	9.0	10.0	11.4	12.3	13.0	13.6	14.2	14.8	15.7	16.8	17.7
70–74	3,812	8.3	9.4	10.8	11.7	12.3	13.0	13.6	14.2	14.9	16.1	17.2
75–79	2,616	7.6	8.5	9.7	10.7	11.4	12.0	12.7	13.4	14.2	15.2	16.0
80–84	1,564	6.9	7.9	9.0	10.0	10.7	11.4	12.0	12.7	13.5	14.5	15.5
85–89	620	5.9	6.9	8.1	8.7	9.3	9.9	10.4	11.4	12.2	13.4	14.5
90 +	164	5.1	6.9	7.9	8.5	9.3	9.7	10.5	10.9	11.5	12.1	14.0
Central and Eastern Europe (pooled *n* = 6,271)												
50–54	239	8.3	10.0	11.4	12.6	13.3	14.3	15.1	15.6	16.9	18.4	19.4
55–59	889	9.4	10.2	11.7	12.9	13.8	14.5	15.0	15.6	16.7	17.9	18.9
60–64	1,245	8.7	10.2	11.4	12.2	13.0	13.6	14.4	15.2	16.0	17.2	18.1
65–69	1,402	8.2	9.7	11.1	11.8	12.7	13.4	14.1	14.8	15.9	16.9	18.0
70–74	1,273	7.8	9.2	10.4	11.3	11.8	12.4	13.2	13.9	14.5	15.7	16.8
75–79	701	6.6	8.1	9.5	10.4	11.2	12.0	12.7	13.3	14.2	15.2	16.3
80–84	358	7.0	7.7	9.0	9.8	10.6	11.0	11.7	12.4	13.4	14.9	16.2
85–89	135	6.9	7.3	8.4	9.2	9.7	10.4	11.3	12.3	13.7	15.9	15.9
90 +	29	4.7	5.8	6.9	7.8	8.5	8.8	8.9	10.0	11.4	11.9	12.2
Northern Europe (pooled *n* = 4,173)												
50–54	224	10.0	12.0	13.7	14.8	15.5	16.0	16.6	17.3	18.3	19.0	20.5
55–59	541	10.8	11.6	12.6	13.6	14.2	14.8	15.6	16.5	17.0	17.9	19.2
60–64	730	9.3	11.5	12.6	13.3	13.9	14.6	15.4	16.1	17.1	18.1	19.0
65–69	758	9.2	10.5	11.7	12.6	13.3	13.7	14.3	15.1	15.8	17.2	18.2
70–74	761	9.0	10.3	11.4	12.3	12.9	13.3	13.9	14.5	15.2	16.5	17.3
75–79	605	8.6	9.5	10.5	11.2	11.9	12.4	13.0	13.6	14.2	15.2	16.1
80–84	378	7.9	8.8	9.7	10.5	11.0	11.4	12.0	12.6	13.4	14.4	15.5
85–89	136	7.0	7.6	9.0	9.3	9.6	10.1	10.5	11.2	12.2	13.0	15.1
90 +	40	5.1	6.0	7.7	9.0	9.5	9.6	10.1	10.5	11.6	13.2	13.2
Southern Europe (pooled *n* = 3,165)												
50–54	77	8.2	11.5	12.1	13.1	13.4	14.0	14.9	15.5	15.7	16.5	17.9
55–59	328	9.7	9.9	11.8	12.2	13.2	13.9	14.7	15.0	15.7	17.2	18.0
60–64	510	8.7	8.9	10.8	12.1	12.5	13.3	14.2	15.0	16.0	17.4	18.0
65–69	662	8.1	9.1	10.2	11.4	12.2	13.0	13.6	14.3	14.9	16.3	17.3
70–74	617	7.9	8.8	9.9	11.1	11.8	12.5	13.3	13.9	14.7	16.0	17.2
75–79	506	7.1	8.1	9.0	9.9	10.7	11.5	12.1	12.8	13.7	14.9	15.6
80–84	299	6.1	7.0	8.2	9.2	10.2	10.9	11.7	12.4	13.1	14.0	15.2
85–89	141	5.0	5.9	7.5	8.3	9.0	9.7	10.4	11.3	11.8	13.3	14.5
90 +	25	4.4	7.4	7.9	8.6	9.7	10.2	10.6	10.8	10.9	11.5	12.9
Western Europe (pooled *n* = 5,938)												
50–54	310	11.0	11.5	12.9	13.6	14.7	15.3	16.1	16.7	17.4	18.3	18.9
55–59	709	9.7	11.0	12.4	13.3	13.9	14.6	15.3	16.2	16.9	17.9	18.9
60–64	1,007	9.9	11.4	12.6	13.4	14.2	14.9	15.4	15.8	16.7	17.5	18.4
65–69	1,140	9.8	10.8	12.0	12.8	13.3	13.9	14.5	15.1	15.9	16.9	17.7
70–74	1,161	8.8	10.2	11.3	12.1	12.7	13.4	13.9	14.5	15.2	16.4	17.4
75–79	804	8.0	9.0	10.3	11.0	11.9	12.4	13.0	13.6	14.5	15.5	16.1
80–84	529	7.8	8.6	9.5	10.5	11.2	11.7	12.5	13.0	13.7	14.7	15.6
85–89	208	6.2	7.1	8.1	8.8	9.3	10.0	10.4	11.4	12.1	12.9	13.7
90 +	70	5.9	6.9	8.1	8.7	9.3	10.2	10.9	11.4	12.1	14.0	14.4

The following classification of countries to European regions was used: Central and Eastern Europe (Bulgaria, Croatia, Czech Republic, Hungary, Poland, Romania, Slovakia, and Slovenia); Northern Europe (Denmark, Estonia, Finland, Latvia, Lithuania, and Sweden); Southern Europe (Cyprus, Greece, Italy, Malta, Portugal, and Spain); and Western Europe (Austria, Belgium, France, Germany, Luxembourg, Netherlands, and Switzerland)

### Reference values for handgrip strength in the standing position by European regions

#### Absolute handgrip strength

Compared to the pan-European percentiles, reference values for handgrip strength among women in Central and Eastern Europe were higher in 15, equal in 53, and lower in 31 out of 99 percentile-by-age comparisons (R_d_: −3 to 2 kg). Compared to the pan-European percentiles, reference values for handgrip strength among men in Central and Eastern Europe were higher in 14, equal in 19, and lower in 66 out of 99 percentile-by-age comparisons (R_d_: −3 to 6 kg).

Compared to the pan-European percentiles, reference values for handgrip strength among women in Northern Europe were higher in 90 and equal in 9 out of 99 percentile-by-age comparisons (R_d_: 0 to 5 kg). Compared to the pan-European percentiles, reference values for handgrip strength among men in Northern Europe were higher in 98 and equal in 1 out of 99 percentile-by-age comparisons (R_d_: 0 to 7 kg).

Compared to the pan-European percentiles, reference values for handgrip strength among women in Southern Europe were higher in 2, equal in 17, and lower in 80 out of 99 percentile-by-age comparisons (R_d_: −6 to 1 kg). Compared to the pan-European percentiles, reference values for handgrip strength among men in Southern Europe were higher in 6, equal in 6, and lower in 87 out of 99 percentile-by-age comparisons (R_d_: −7 to 3 kg).

Compared to the pan-European percentiles, reference values for handgrip strength among women in Western Europe were higher in 52, equal in 42, and lower in 5 out of 99 percentile-by-age comparisons (R_d_: −1 to 3 kg). Compared to the pan-European percentiles, reference values for handgrip strength among men in Western Europe were higher in 94, equal in 4, and lower in 1 out of 99 percentile-by-age comparisons (R_d_: −1 to 6 kg).

#### Relative handgrip strength

Compared to the pan-European percentiles, reference values for relative handgrip strength among women in Central and Eastern Europe were higher in 37, equal in 16, and lower in 46 out of 99 percentile-by-age comparisons (R_d_: −1.3 to 0.8 kg/m^2^). Compared to the pan-European percentiles, reference values for relative handgrip strength among men in Central and Eastern Europe were higher in 26, equal in 8, and lower in 65 out of 99 percentile-by-age comparisons (R_d_: −1.8 to 2.5 kg/m^2^).

Compared to the pan-European percentiles, reference values for relative handgrip strength among women in Northern Europe were higher in 91, equal in 7, and lower in 1 out of 99 percentile-by-age comparisons (R_d_: −0.2 to 1.6 kg/m^2^). Compared to the pan-European percentiles, reference values for relative handgrip strength among men in Northern Europe were higher in 81, equal in 7, and lower in 11 out of 99 percentile-by-age comparisons (R_d_: −0.9 to 1.6 kg/m^2^).

Compared to the pan-European percentiles, reference values for relative handgrip strength among women in Southern Europe were higher in 9, equal in 15, and lower in 75 out of 99 percentile-by-age comparisons (R_d_: −2.5 to 0.8 kg/m^2^). Compared to the pan-European percentiles, reference values for relative handgrip strength among men in Southern Europe were higher in 6, equal in 5, and lower in 88 out of 99 percentile-by-age comparisons (R_d_: −1.8 to 0.5 kg/m^2^).

Compared to the pan-European percentiles, reference values for handgrip strength among women in Western Europe were higher in 66, equal in 19, and lower in 14 out of 99 percentile-by-age comparisons (R_d_: −0.4 to 1.4 kg/m^2^). Compared to the pan-European percentiles, reference values for relative handgrip strength among men in Western Europe were higher in 85, equal in 10, and lower in 4 out of 99 percentile-by-age comparisons (R_d_: −0.8 to 1.9 kg/m^2^).

### Pan-European reference values for handgrip strength in the sitting position

#### Absolute handgrip strength

For women, the highest handgrip strength was observed among the 50–54-year-olds and 55–59-year-olds (5th percentile = 20 kg; 50th percentile = 29 kg; 95th percentile = 40 kg; Table 7) and the lowest among the ≥ 90-year-olds (5th percentile = 6 kg; 50th percentile = 14 kg; 95th percentile = 24 kg). For men, the highest handgrip strength was observed among the 50–54-year-olds and 60–64-year-olds (5th percentile = 29 kg; 50th percentile = 46 kg; 95th percentile = 60 kg; Table 8) and the lowest among the ≥ 90-year-olds (5th percentile = 10 kg; 50th percentile = 25 kg; 95th percentile = 37 kg). Compared with women, men had higher handgrip strength in all 99 percentile-by-age comparisons (R_d_: 4 to 23 kg).

**Table 7:** Reference values for absolute handgrip strength in the sitting position for women

Age (years)	*n*	Weighted percentile (kg)										
		5th	10th	20th	30th	40th	50th	60th	70th	80th	90th	95th
Europe (pooled *n* = 8,207)												
50–54	409	20	21	24	26	27	28	30	32	34	38	40
55–59	919	18	20	24	25	28	29	30	32	34	35	37
60–64	1,129	12	18	22	24	25	27	28	30	32	36	38
65–69	1,325	15	17	20	22	24	25	27	29	31	33	35
70–74	1,391	14	16	19	20	22	23	25	27	29	31	34
75–79	1,206	12	14	16	18	20	21	23	24	27	30	32
80–84	989	11	13	15	17	18	20	21	23	25	28	29
85–89	565	8	10	13	14	15	17	18	20	23	25	29
90 +	274	6	8	10	12	13	14	15	16	18	20	24
Central and Eastern Europe (pooled *n* = 2,779)												
50–54	142	15	18	24	26	27	29	30	31	32	35	40
55–59	307	16	20	25	26	29	30	32	32	33	34	37
60–64	376	13	17	19	21	24	25	27	28	31	33	36
65–69	498	12	16	20	22	24	25	26	29	30	33	35
70–74	525	11	14	18	19	20	23	24	26	28	31	35
75–79	383	10	12	15	18	19	21	22	24	27	30	32
80–84	307	10	11	15	16	18	20	21	22	24	28	30
85–89	173	10	11	12	13	15	17	18	19	23	26	29
90 +	68	4	7	10	12	14	14	16	18	20	20	26
Northern Europe (pooled *n* = 1,708)												
50–54	87	23	26	26	27	30	30	34	35	35	38	42
55–59	169	17	20	25	27	30	30	32	32	34	35	38
60–64	196	19	21	23	25	27	29	31	33	35	37	38
65–69	234	14	18	20	22	24	26	28	29	31	34	38
70–74	256	14	17	21	23	24	25	26	28	30	32	36
75–79	254	12	15	17	20	21	22	23	25	28	30	34
80–84	259	12	13	15	17	19	20	22	24	25	28	30
85–89	175	7	10	12	15	17	18	19	21	23	26	31
90 +	78	7	10	11	12	13	14	15	17	19	24	24
Southern Europe (pooled *n* = 1,409)												
50–54	49	21	21	24	24	25	26	27	29	29	34	35
55–59	151	16	20	20	24	25	26	28	30	33	35	35
60–64	208	18	20	22	23	25	25	27	28	28	30	35
65–69	234	11	15	17	20	22	24	25	27	30	33	34
70–74	243	14	16	18	20	21	23	23	25	27	29	30
75–79	254	10	12	15	15	17	19	20	21	23	27	31
80–84	152	11	13	15	16	17	18	20	22	23	28	30
85–89	75	7	9	12	14	15	17	18	20	22	25	29
90 +	43	8	8	10	10	12	12	13	14	15	18	18
Western Europe (pooled *n* = 2,311)												
50–54	131	20	21	22	27	28	31	31	34	38	40	40
55–59	292	20	22	25	27	29	30	31	33	34	37	39
60–64	349	12	18	23	25	27	28	30	32	34	38	40
65–69	359	18	19	22	24	25	26	28	30	31	34	36
70–74	367	16	18	20	22	23	25	27	28	31	33	35
75–79	315	15	17	19	20	22	24	25	27	28	30	32
80–84	271	11	13	16	17	18	20	22	23	25	28	28
85–89	142	9	11	13	15	16	17	19	20	23	25	29
90 +	85	5	10	12	13	14	15	16	16	20	23	25

The following classification of countries to European regions was used: Central and Eastern Europe (Bulgaria, Croatia, Czech Republic, Hungary, Poland, Romania, Slovakia, and Slovenia); Northern Europe (Denmark, Estonia, Finland, Latvia, Lithuania, and Sweden); Southern Europe (Cyprus, Greece, Italy, Malta, Portugal, and Spain); and Western Europe (Austria, Belgium, France, Germany, Luxembourg, Netherlands, and Switzerland)

**Table 8:** Reference values for absolute handgrip strength in the sitting position for men

Age (years)	*n*	Weighted percentile (kg)										
		5th	10th	20th	30th	40th	50th	60th	70th	80th	90th	95th
Europe (pooled *n* = 6,088)												
50–54	226	28	33	38	40	41	44	45	48	52	57	59
55–59	598	29	34	37	40	43	45	47	50	53	56	60
60–64	934	27	30	38	40	44	46	47	48	51	56	59
65–69	1,140	26	30	34	37	39	41	43	45	50	53	55
70–74	1,103	24	27	31	34	36	39	41	43	46	50	52
75–79	899	19	23	27	30	33	35	37	39	42	45	48
80–84	641	17	21	25	28	30	32	34	36	38	41	45
85–89	378	16	19	22	24	27	29	30	33	36	39	41
90 +	169	10	15	20	21	23	25	27	29	32	35	37
Central and Eastern Europe (pooled *n* = 1,900)												
50–54	73	25	25	25	36	42	44	45	47	52	60	61
55–59	175	16	28	35	38	44	45	45	48	51	53	56
60–64	324	10	27	32	36	40	43	45	48	50	53	59
65–69	384	20	26	33	34	36	38	39	42	45	50	52
70–74	370	16	21	27	32	35	36	38	41	45	48	50
75–79	260	15	16	26	30	31	34	36	38	40	44	47
80–84	169	13	15	19	25	28	30	33	36	39	42	50
85–89	112	15	15	17	20	25	28	31	33	35	37	40
90 +	33	14	16	18	19	20	20	28	29	29	32	32
Northern Europe (pooled *n* = 1,085)												
50–54	57	32	37	44	44	45	45	50	53	54	55	59
55–59	109	34	38	42	44	47	50	51	54	57	65	65
60–64	154	25	29	37	40	41	45	51	53	56	61	63
65–69	184	24	32	35	37	40	43	45	47	50	53	57
70–74	171	24	27	33	37	39	42	45	46	49	52	63
75–79	137	25	29	31	34	35	37	40	43	46	49	53
80–84	135	19	23	28	30	30	33	37	39	41	49	51
85–89	89	18	21	24	26	26	28	31	35	37	43	43
90 +	49	10	16	18	21	23	25	26	28	33	43	43
Southern Europe (pooled *n* = 1,127)												
50–54	15	37	37	40	41	41	44	46	46	48	57	62
55–59	91	30	30	35	37	39	40	43	46	47	50	53
60–64	158	25	30	38	41	43	45	48	48	48	52	55
65–69	217	25	29	31	34	37	38	40	42	45	51	52
70–74	211	23	25	28	30	34	36	38	42	44	50	51
75–79	199	19	22	26	30	30	34	35	36	39	42	45
80–84	139	20	21	23	26	28	30	31	34	36	39	40
85–89	68	16	17	19	22	26	29	30	30	33	37	41
90 +	29	8	8	12	21	23	24	25	26	27	35	36
Western Europe (pooled *n* = 1,976)												
50–54	81	33	33	38	39	41	42	45	48	52	55	58
55–59	223	34	35	40	42	46	49	51	53	55	59	61
60–64	298	30	34	40	43	45	46	48	51	55	58	61
65–69	355	29	32	37	39	42	43	45	49	52	55	56
70–74	351	27	30	34	35	38	40	43	45	47	50	53
75–79	303	23	24	28	32	34	36	38	40	43	47	49
80–84	198	19	24	28	30	32	33	36	38	39	42	45
85–89	109	19	22	23	26	28	29	30	34	37	40	41
90 +	58	15	16	20	21	25	27	29	30	34	36	39

The following classification of countries to European regions was used: Central and Eastern Europe (Bulgaria, Croatia, Czech Republic, Hungary, Poland, Romania, Slovakia, and Slovenia); Northern Europe (Denmark, Estonia, Finland, Latvia, Lithuania, and Sweden); Southern Europe (Cyprus, Greece, Italy, Malta, Portugal, and Spain); and Western Europe (Austria, Belgium, France, Germany, Luxembourg, Netherlands, and Switzerland)

#### Relative handgrip strength

For women, the highest relative handgrip strength was observed among the 50–54-year-olds and 55–59-year-olds (5th percentile = 7.1 kg/m^2^; 50th percentile = 10.8 kg/m^2^; 95th percentile = 14.8 kg/m^2^; Table 9) and the lowest among the ≥ 90-year-olds (5th percentile = 2.6 kg/m^2^; 50th percentile = 5.5 kg/m^2^; 95th percentile = 9.6 kg/m^2^). For men, the highest relative handgrip strength was observed among the 50–54-year-olds and 60–64-year-olds (5th percentile = 9.1 kg/m^2^; 50th percentile = 14.9 kg/m^2^; 95th percentile = 18.8 kg/m^2^; Table 10) and the lowest among the ≥ 90-year-olds (5th percentile = 3.8 kg/m^2^; 50th percentile = 8.2 kg/m^2^; 95th percentile = 12.9 kg/m^2^). Compared with women, men had higher relative handgrip strength in all 99 percentile-by-age comparisons (R_d_: 1.2 to 5.3 kg/m^2^).

**Table 9:** Reference values for relative handgrip strength in the sitting position for women

Age (years)	*n*	Weighted percentile (kg/m^2^)										
		5th	10th	20th	30th	40th	50th	60th	70th	80th	90th	95th
Europe (pooled *n* = 8,103)												
50–54	405	6.8	7.4	8.6	9.5	10.2	10.7	11.1	11.7	12.4	13.3	14.8
55–59	914	7.1	7.8	8.7	9.5	10.3	10.8	11.1	11.6	12.3	13.1	13.5
60–64	1,120	4.6	6.9	8.2	9.2	9.6	10.2	10.7	11.5	12.0	13.1	13.8
65–69	1,317	5.5	6.6	7.6	8.3	9.1	9.7	10.2	10.6	11.4	12.4	13.2
70–74	1,378	5.5	6.2	7.3	7.8	8.4	8.9	9.5	10.0	11.0	12.0	12.6
75–79	1,187	4.6	5.2	6.3	7.0	7.5	8.2	8.8	9.3	10.2	11.0	11.9
80–84	967	4.1	4.9	6.0	6.5	7.0	7.4	8.0	8.7	9.3	10.3	11.3
85–89	551	3.2	4.0	4.9	5.5	6.0	6.6	7.1	7.9	8.8	9.8	10.6
90 +	264	2.6	3.5	4.3	4.4	5.1	5.5	5.8	6.4	6.9	7.6	9.6
Central and Eastern Europe (pooled *n* = 2,745)												
50–54	141	5.2	6.4	8.9	9.2	9.7	10.1	10.6	11.2	11.6	12.9	14.5
55–59	304	6.2	7.5	8.8	9.9	10.3	11.0	11.1	11.1	12.2	13.3	13.3
60–64	375	5.0	6.0	7.1	8.0	8.6	9.3	9.9	10.4	11.0	12.2	13.3
65–69	491	4.7	5.9	7.3	8.2	8.8	9.5	10.0	10.6	11.4	12.1	12.7
70–74	521	4.1	5.5	6.6	7.3	7.8	8.4	9.1	9.8	10.7	12.1	13.0
75–79	380	3.7	4.9	5.9	6.6	7.3	8.1	8.7	9.2	10.3	11.8	12.5
80–84	302	4.0	4.4	5.6	6.4	7.0	7.5	8.1	8.5	9.3	10.7	11.9
85–89	166	3.5	4.0	4.9	5.4	5.6	6.4	6.9	7.6	9.3	10.0	11.0
90 +	65	1.5	2.7	3.8	4.9	5.5	5.6	6.5	6.9	7.3	7.7	10.4
Northern Europe (pooled *n* = 1,702)												
50–54	87	8.6	10.2	10.3	10.3	10.8	11.3	12.1	12.1	12.9	13.6	14.2
55–59	169	5.6	7.4	8.9	9.8	10.1	10.8	11.8	12.0	12.2	12.6	14.5
60–64	194	6.3	7.6	8.6	9.4	10.0	10.6	11.1	11.8	12.6	13.2	14.2
65–69	234	5.8	6.6	7.5	8.2	9.0	9.7	10.0	10.5	11.5	12.5	14.2
70–74	255	5.3	6.4	8.0	8.6	9.1	9.4	9.8	10.4	11.1	12.1	13.4
75–79	253	4.7	5.3	6.5	7.4	7.8	8.2	8.8	9.7	10.3	11.2	12.5
80–84	257	4.5	5.2	5.9	6.2	7.2	7.9	8.3	9.0	9.6	10.8	12.2
85–89	175	2.5	4.1	4.8	5.6	6.2	6.9	7.4	7.9	8.7	10.0	11.1
90 +	78	3.2	3.7	4.5	4.9	5.0	5.3	6.0	6.8	7.8	9.4	9.6
Southern Europe (pooled *n* = 1,358)												
50–54	48	7.5	7.5	8.6	9.5	9.7	10.4	10.9	11.1	11.7	13.3	13.3
55–59	151	5.5	7.8	8.3	9.1	9.4	10.2	10.7	11.4	12.0	13.1	13.4
60–64	205	6.9	7.7	9.0	9.5	9.8	10.5	10.5	11.3	12.0	12.0	12.9
65–69	233	4.5	5.5	6.7	8.0	8.7	9.4	10.1	10.4	11.2	13.4	13.4
70–74	235	5.8	6.2	7.4	7.8	8.2	8.7	9.2	9.8	10.7	11.7	12.0
75–79	239	4.2	4.9	5.6	6.5	7.0	7.3	8.0	8.6	9.2	10.0	11.5
80–84	139	4.3	5.1	6.1	6.4	6.7	7.3	8.0	8.8	9.5	10.3	11.7
85–89	70	3.1	3.9	4.9	5.5	5.9	6.1	6.9	8.1	8.4	9.8	10.9
90 +	38	3.2	3.2	3.9	4.4	4.4	4.4	5.2	5.8	6.3	6.8	7.1
Western Europe (pooled *n* = 2,298)												
50–54	129	7.0	7.4	8.2	9.7	10.4	11.0	11.4	12.0	12.7	14.3	14.9
55–59	290	7.4	8.0	9.2	9.9	10.4	11.1	11.4	11.7	12.4	13.1	13.7
60–64	346	4.6	7.5	8.2	9.2	9.8	10.5	11.2	11.7	12.6	13.6	14.2
65–69	359	6.8	7.1	8.0	8.8	9.4	9.9	10.4	11.0	11.6	12.4	13.0
70–74	367	5.9	6.5	7.6	8.2	8.8	9.3	9.9	10.7	11.3	12.1	12.9
75–79	315	5.7	6.4	7.1	7.8	8.5	9.0	9.4	10.0	10.6	11.2	12.1
80–84	269	4.1	4.9	6.0	6.5	7.0	7.5	7.9	8.7	9.1	10.0	11.1
85–89	140	3.6	4.0	5.2	5.8	6.5	6.9	7.2	7.7	8.8	9.7	10.4
90 +	83	2.1	3.7	4.4	5.2	5.4	5.7	6.2	6.7	7.1	9.4	10.0

The following classification of countries to European regions was used: Central and Eastern Europe (Bulgaria, Croatia, Czech Republic, Hungary, Poland, Romania, Slovakia, and Slovenia); Northern Europe (Denmark, Estonia, Finland, Latvia, Lithuania, and Sweden); Southern Europe (Cyprus, Greece, Italy, Malta, Portugal, and Spain); and Western Europe (Austria, Belgium, France, Germany, Luxembourg, Netherlands, and Switzerland)

**Table 10:** Reference values for relative handgrip strength in the sitting position for men

Age (years)	*n*	Weighted percentile (kg/m^2^)										
		5th	10th	20th	30th	40th	50th	60th	70th	80th	90th	95th
Europe (pooled *n* = 6,044)												
50–54	225	9.1	9.4	11.9	12.6	13.4	14.2	14.9	15.5	16.2	18.0	18.7
55–59	596	8.8	10.6	12.1	12.9	13.7	14.5	15.4	16.1	17.2	18.0	18.8
60–64	926	9.0	10.4	12.1	13.4	14.1	14.9	15.2	16.0	16.6	17.8	18.6
65–69	1,135	8.7	9.7	11.2	11.9	12.8	13.3	14.0	14.9	15.6	16.9	17.6
70–74	1,092	7.9	9.0	10.3	11.1	11.9	12.6	13.4	14.0	15.1	16.1	17.4
75–79	892	6.6	7.9	9.0	9.8	10.8	11.7	12.2	12.9	13.7	15.1	15.6
80–84	637	6.0	7.3	8.6	9.4	10.0	10.5	11.2	11.9	12.8	13.5	14.9
85–89	374	5.9	6.6	7.4	7.9	9.2	9.8	10.3	10.8	12.1	13.1	13.5
90 +	167	3.8	5.1	6.6	6.9	7.8	8.2	9.5	9.8	10.7	11.8	12.9
Central and Eastern Europe (pooled *n* = 1,891)												
50–54	73	7.4	8.2	8.2	12.8	13.8	14.2	14.9	15.2	16.5	18.1	20.8
55–59	175	5.2	8.6	11.3	12.9	13.8	14.3	15.6	16.1	16.6	17.6	18.3
60–64	322	3.3	8.7	10.4	12.2	13.1	14.2	14.9	15.4	16.0	17.7	19.6
65–69	383	6.7	9.0	10.4	11.4	11.8	12.9	13.4	13.7	14.8	16.1	17.1
70–74	366	5.1	7.4	9.4	10.4	11.3	12.1	12.8	13.5	14.5	16.0	17.2
75–79	260	4.8	5.4	8.7	9.7	10.4	11.1	11.9	12.4	13.4	14.5	15.8
80–84	168	5.0	5.3	6.8	8.3	9.2	10.1	11.0	12.1	12.9	14.5	16.7
85–89	111	5.2	5.3	6.2	7.0	8.5	9.5	10.3	10.9	11.8	13.0	13.7
90 +	33	5.6	5.7	6.7	6.9	6.9	6.9	9.6	10.6	10.7	10.9	10.9
Northern Europe (pooled *n* = 1,084)												
50–54	57	9.9	11.3	12.6	12.6	13.5	14.1	14.2	16.6	17.7	18.3	18.4
55–59	109	10.1	11.0	12.1	13.2	14.7	15.2	15.6	16.7	17.7	18.8	20.2
60–64	153	8.2	9.4	11.9	12.4	13.6	14.6	15.5	16.5	17.3	18.1	18.7
65–69	184	7.6	10.5	11.3	11.9	12.7	13.1	13.8	14.8	15.8	16.4	17.5
70–74	171	7.5	9.0	10.6	11.7	12.8	13.7	14.0	14.5	15.1	16.4	18.6
75–79	137	7.8	9.0	9.9	10.9	11.4	12.0	12.8	13.9	14.5	15.2	15.8
80–84	135	6.6	7.5	8.9	9.4	10.2	10.8	11.6	12.3	12.9	15.4	15.8
85–89	89	5.8	6.9	7.9	8.4	8.8	9.4	10.3	11.3	12.3	13.1	13.7
90 +	49	4.4	5.1	6.5	6.9	7.6	8.3	8.7	9.2	10.8	14.0	14.0
Southern Europe (pooled *n* = 1,101)												
50–54	14	11.7	11.7	13.0	13.0	13.4	14.2	15.5	15.6	16.0	18.6	19.6
55–59	90	9.5	10.2	11.4	11.8	12.7	13.3	13.9	14.7	15.5	17.7	19.0
60–64	154	8.8	10.4	12.8	13.5	14.7	15.1	15.9	16.6	16.6	17.6	17.8
65–69	215	9.1	9.6	10.7	11.8	12.6	13.2	13.6	14.9	15.4	16.3	17.3
70–74	205	7.9	8.6	9.3	10.4	11.2	11.9	12.6	13.7	14.7	16.8	18.2
75–79	194	6.6	7.2	8.8	9.5	11.0	11.7	12.2	12.9	13.4	15.2	15.6
80–84	136	7.3	7.6	8.5	9.3	9.9	10.4	11.0	11.4	12.3	13.2	13.6
85–89	65	5.6	6.1	6.8	7.6	9.1	10.0	10.4	10.7	12.6	12.9	13.1
90 +	28	2.9	3.0	5.1	7.2	7.8	8.1	9.1	9.6	10.1	12.0	12.9
Western Europe (pooled *n* = 1,968)												
50–54	81	9.4	9.4	11.5	12.6	13.1	13.9	14.7	15.4	16.8	17.7	18.8
55–59	222	10.7	12.0	13.0	13.6	14.5	15.2	15.8	16.7	17.5	18.1	18.6
60–64	297	9.3	11.0	12.7	13.5	14.1	14.8	15.2	15.9	16.8	18.0	18.8
65–69	353	9.3	10.4	11.7	12.4	13.1	13.8	14.7	15.3	16.1	17.2	18.1
70–74	350	8.5	9.8	10.8	11.8	12.3	13.2	13.7	14.5	15.4	16.1	17.3
75–79	301	7.5	8.3	9.1	10.1	10.9	11.5	12.2	13.0	14.0	15.2	15.8
80–84	198	6.7	7.9	8.9	9.6	10.3	10.8	11.4	12.4	12.9	13.7	15.0
85–89	109	6.6	7.1	7.8	8.6	9.4	9.8	10.3	10.9	12.3	13.4	13.8
90 +	57	4.9	5.3	6.7	7.4	8.1	9.3	9.7	10.7	10.7	12.1	14.5

The following classification of countries to European regions was used: Central and Eastern Europe (Bulgaria, Croatia, Czech Republic, Hungary, Poland, Romania, Slovakia, and Slovenia); Northern Europe (Denmark, Estonia, Finland, Latvia, Lithuania, and Sweden); Southern Europe (Cyprus, Greece, Italy, Malta, Portugal, and Spain); and Western Europe (Austria, Belgium, France, Germany, Luxembourg, Netherlands, and Switzerland)

### Reference values for handgrip strength in the sitting position according to European regions

#### Absolute handgrip strength

Compared to the pan-European percentiles, reference values for handgrip strength among women in Central and Eastern Europe were higher in 17, equal in 37, and lower in 45 out of 99 percentile-by-age comparisons (R_d_: −5 to 2 kg). Compared to the pan-European percentiles, reference values for handgrip strength among men in Central and Eastern Europe were higher in 11, equal in 10, and lower in 78 out of 99 percentile-by-age comparisons (R_d_: −17 to 5 kg).

Compared to the pan-European percentiles, reference values for handgrip strength among women in Northern Europe were higher in 67, equal in 28, and lower in 4 out of 99 percentile-by-age comparisons (R_d_: −1 to 7 kg). Compared to the pan-European percentiles, reference values for handgrip strength among men in Northern Europe were higher in 75, equal in 12, and lower in 12 out of 99 percentile-by-age comparisons (R_d_: −3 to 11 kg).

Compared to the pan-European percentiles, reference values for handgrip strength among women in Southern Europe were higher in 5, equal in 24, and lower in 70 out of 99 percentile-by-age comparisons (R_d_: −6 to 6 kg). Compared to the pan-European percentiles, reference values for handgrip strength among men in Southern Europe were higher in 10, equal in 17, and lower in 72 out of 99 percentile-by-age comparisons (R_d_: −8 to 9 kg).

Compared to the pan-European percentiles, reference values for handgrip strength among women in Western Europe were higher in 72, equal in 24, and lower in 3 out of 99 percentile-by-age comparisons (R_d_: −2 to 4 kg). Compared to the pan-European percentiles, reference values for handgrip strength among men in Western Europe were higher in 81, equal in 14, and lower in 4 out of 99 percentile-by-age comparisons (R_d_: −2 to 5 kg).

#### Relative handgrip strength

Compared to the pan-European percentiles, reference values for relative handgrip strength among women in Central and Eastern Europe were higher in 27, equal in 8, and lower in 64 out of 99 percentile-by-age comparisons (R_d_: −1.6 to 0.8 kg/m^2^). Compared to the pan-European percentiles, reference values for relative handgrip strength among men in Central and Eastern Europe were higher in 20, equal in 7, and lower in 72 out of 99 percentile-by-age comparisons (R_d_: −5.7 to 2.1 kg/m^2^).

Compared to the pan-European percentiles, reference values for relative handgrip strength among women in Northern Europe were higher in 73, equal in 7, and lower in 19 out of 99 percentile-by-age comparisons (R_d_: −1.5 to 2.8 kg/m^2^). Compared to the pan-European percentiles, reference values for relative handgrip strength among men in Northern Europe were higher in 65, equal in 10, and lower in 24 out of 99 percentile-by-age comparisons (R_d_: −1.1 to 2.2 kg/m^2^).

Compared to the pan-European percentiles, reference values for relative handgrip strength among women in Southern Europe were higher in 21, equal in 14, and lower in 64 out of 99 percentile-by-age comparisons (R_d_: −2.5 to 2.3 kg/m^2^). Compared to the pan-European percentiles, reference values for relative handgrip strength among men in Southern Europe were higher in 28, equal in 13, and lower in 58 out of 99 percentile-by-age comparisons (R_d_: −2.1 to 2.6 kg/m^2^).

Compared to the pan-European percentiles, reference values for handgrip strength among women in Western Europe were higher in 75, equal in 14, and lower in 10 out of 99 percentile-by-age comparisons (R_d_: −0.5 to 1.8 kg/m^2^). Compared to the pan-European percentiles, reference values for relative handgrip strength among men in Western Europe were higher in 79, equal in 9, and lower in 11 out of 99 percentile-by-age comparisons (R_d_: −0.4 to 1.9 kg/m^2^).

### Differences between reference values for handgrip strength in the standing and sitting positions

#### Absolute handgrip strength

Compared to pan-European percentiles for handgrip strength in the sitting position, reference values for handgrip strength in the standing position were higher in 69, equal in 21, and lower in 9 out of 99 percentile-by-age comparisons among women (R_d_: −2 to 7 kg). Compared to pan-European percentiles for handgrip strength in the sitting position, reference values for handgrip strength in the standing position were higher in 65, equal in 22, and lower in 12 out of 99 percentile-by-age comparisons among men (R_d_: −2 to 3 kg kg/m^2^).

#### Relative handgrip strength

Compared to pan-European percentiles for handgrip strength in the sitting position, reference values for handgrip strength in the standing position were higher in 83, equal in 8, and lower in 8 out of 99 percentile-by-age comparisons among women (R_d_: −0.7 to 2.4 kg/m^2^). Compared to pan-European percentiles for handgrip strength in the sitting position, reference values for handgrip strength in the standing position were higher in 74, equal in 4, and lower in 21 out of 99 percentile-by-age comparisons among men (R_d_: −0.9 to 1.9 kg/m^2^).

### Analyses of combined data for handgrip strength in the standing and sitting positions

Reference values calculated from the data collected using handgrip test in the standing and sitting positions combined are presented as follows: Supplementary Table 1 for female absolute handgrip strength, Supplementary Table 2 for male absolute handgrip strength, Supplementary Table 3 for female relative handgrip strength, and Supplementary Table 4 for male relative handgrip strength.

## Discussion

### Key findings

The key findings of this study are: (a) reference values for handgrip strength tend to be lower in older age groups, generally showing the highest handgrip strength among the 50–54-year-olds and the lowest among the 90 + year-olds; (b) across all age groups, reference values for handgrip strength are higher among men than among women; (c) reference values for handgrip strength tend to be higher in Northern and Western Europe, compared with Southern and Central/Eastern Europe, even when adjusted for body height. Additionally, participants who performed the test standing generally showed greater handgrip strength than those tested in the sitting position. These reference values, stratified by sex, age, European region, and standing/sitting position, can facilitate benchmarking for clinical, population health, and research purposes.

### Comparison with previous findings

While there is a lack of studies establishing reference values for handgrip strength in Europe, a recent study provided international reference values using data from 69 countries/regions [15]. This was the largest and geographically most comprehensive study on reference values for handgrip strength to date, and it included data from various European countries. However, given that the reference values were not provided specifically for Europe or individual European countries, we could only compare our results with their international findings. While some percentiles in our study were fairly similar to the international reference values, others were considerably higher or lower. For instance, the 95th percentile for handgrip strength in the sitting position for males aged 70–74 years from Northern Europe exceeded the international reference value by 9 kg. In contrast, the 95th percentile for men aged 55–59 years from Southern Europe in the current study was 9 kg lower, compared to the respective reference value in the international study. These results show the importance of establishing region-specific reference values for handgrip strength. However, it should be noted that despite lower generalizability, the international reference values can still serve as a useful fallback in the absence of regional data.

Despite the above-mentioned difference in percentiles, findings on sex and age differences in our study are similar as in the international study [15], suggesting that these demographic variables are important correlates of handgrip strength, regardless of the world region. Furthermore, our findings on sex, age, and North–South differences in handgrip strength are in accordance with previous results for 11 European countries based on data from 2004 [18], suggesting that these demographic and geographic correlates of handgrip strength have been stable over time. However, a study that analysed data from Germany, Spain, and Sweden, collected from 2004 to 2013 found some changes in handgrip strength over time [19], which indicates that reference values determined in our study will also eventually need to be updated. To enable this, it is important to keep on collecting handgrip strength data in Europe within the SHARE study.

### Differences in reference values according to age, sex, European region, and testing position

The observed age gradient and differences between sexes in handgrip strength percentiles are in accordance with previous studies [12–17]. The former finding reflects an age-related decline in muscle strength [30], while the latter finding is due to physiological and morphological differences between men and women, particularly in terms of muscle mass, ratio of muscle-fiber types, and neuromuscular factors [31].

Reference data for absolute handgrip strength indicated higher values for Northern and Western Europe, compared with Southern and Central/Eastern Europe. Given that the population from Northern and Western Europe tends to be taller, it could be assumed that differences in height explain the regional variation in handgrip strength [32]. However, the regional differences in handgrip strength reported in this study persisted even after the adjustments for body height. The use of relative handgrip strength (kg/m^2^) confirmed the same north-to-south gradient, indicating that differences in muscular strength across Europe are not solely attributable to height. While our findings do not directly imply what factors underpin this difference in handgrip strength, the prevalence of muscle-strengthening exercise participation across countries could be a contributing factor [31]. In particular, previous findings show a much lower prevalence of regular (≥ 2 days/week) muscle-strengthening exercise in Southern Europe (range: 0.7%−7.4%) compared to Northern Europe (range: 34.1%–51.6%) [33]. Further research should explore environmental, occupational, nutritional, genetic, and other factors that may contribute to regional differences in handgrip strength observed across Europe.

Previous studies have shown that altering the testing position may influence handgrip strength, with greater values generally observed while standing, compared with sitting [34, 35]. Indeed, our analysis showed that handgrip strength values were generally higher among participants who performed the test while standing. While the standardized SHARE protocol for handgrip strength testing was designed to be performed standing, individuals had the option to perform the test while seated. A larger number of participants performed the test standing (*n* = 44,214) compared to sitting (*n* = 14,295). It might be that individuals with poorer health (e.g., greater frailty) chose to perform the test in the sitting position, which could partially explain the differences in reference values between these conditions. Nevertheless, this finding reinforces the need for standardisation in test administration, as position can influence performance outcomes.

### Practical implications

The reference values generated from this dataset have broad applicability across clinical and public health settings. In clinical practice, they can aid in the early identification of individuals at risk for frailty, functional decline, and other adverse health outcomes, by enabling benchmarking against age-, sex-, and region-specific handgrip strength percentiles [2–8]. From a public health perspective, the reference values for handgrip strength can be used as benchmarks in monitoring population-level trends in physical function and when evaluating outcomes of policies and interventions aimed at promoting healthy ageing across European regions. The availability of both absolute and height-adjusted reference values for handgrip strength in standing and sitting positions enables flexibility in their application, depending on the context and purpose.

### Strengths and limitations

Strengths of this study include: (a) the use of data collected in a large, population-representative sample, by applying harmonised protocols across multiple countries; and (b) the calculation of both absolute and relative height-adjusted reference values for handgrip strength, stratified by age, sex, geographic region, and testing position. Despite its strengths, this study has some limitations that should be acknowledged. First, the dataset includes only individuals aged 50 + years, limiting the extrapolation of the reference values to younger populations. Future studies are needed to extend these reference values to younger adults, to enabling benchmarking for all age groups. Second, although the dataset included 27 countries, not all European countries were represented, somewhat limiting the generalizability of our findings.

## Conclusion

This study provides up-to-date, comprehensive reference values for handgrip strength in European adults aged 50 years and older. The reference values are higher for men, compared with women, and tend to be lower in older age groups. The reference values are generally higher in Northern and Western Europe, compared with Southern and Central/Eastern Europe, even when expressed relative to body height. The reference values tend to be higher for handgrip test performed in the standing position, compared with handgrip test in the sitting position. These reference values can be used as benchmarks in the assessment of handgrip strength for clinical, public health, and research purposes. Their use could facilitate early detection of individuals at risk of functional decline, and inform strategies to promote healthy ageing across Europe.

## Supplementary Information

Below is the link to the electronic supplementary material.

Supplementary file 1 (DOCX 23 KB)

Supplementary file 2 (DOCX 22 KB)

Supplementary file 3 (DOCX 23 KB)

Supplementary file 4 (DOCX 23 KB)

## Data Availability

The data are available upon request from the Survey of Health, Ageing and Retirement in Europe (SHARE) website: https://share-eric.eu/
