# Supplementary material for: Reference values for handgrip strength in Europe: analysis of individual participant data from 27 countries
Source: GeroScience. 2025 Oct 1;48(3):4349–71. doi: 10.1007/s11357-025-01919-9 (PMC13355996; doi:10.1007/s11357-025-01919-9)
Supplement: Supplementary file 1 — (DOCX 23 KB) [file 11357_2025_1919_MOESM1_ESM.docx]

**Electronic Supplementary Material Appendix** **S1.** Reference values for absolute handgrip strength among women: combined data for testing in standing and sitting positions

| **Age (years)** | ***n*** | **Weighted percentile** **(kg)** | | | | | | | | | | |
| --- | --- | --- | --- | --- | --- | --- | --- | --- | --- | --- | --- | --- |
|  |  | **5^th^** | **10^th^** | **20^th^** | **30^th^** | **40^th^** | **50^th^** | **60^th^** | **70^th^** | **80^th^** | **90^th^** | **95^th^** |
| Europe (pooled *n* = 32,801) | | | | | | | | | | | | |
| 50–54 | 1,936 | 19 | 21 | 24 | 26 | 28 | 29 | 30 | 32 | 34 | 37 | 39 |
| 55–59 | 4,328 | 19 | 21 | 24 | 25 | 27 | 29 | 30 | 32 | 33 | 36 | 38 |
| 60–64 | 5,638 | 18 | 21 | 23 | 25 | 26 | 28 | 29 | 31 | 32 | 35 | 37 |
| 65–69 | 6,156 | 16 | 19 | 22 | 24 | 25 | 26 | 27 | 29 | 31 | 34 | 35 |
| 70–74 | 5,780 | 15 | 17 | 20 | 21 | 23 | 24 | 26 | 28 | 29 | 32 | 34 |
| 75–79 | 4,283 | 13 | 15 | 18 | 19 | 21 | 22 | 24 | 25 | 27 | 30 | 32 |
| 80–84 | 2,871 | 12 | 14 | 16 | 18 | 20 | 21 | 22 | 24 | 25 | 28 | 30 |
| 85–89 | 1,312 | 9 | 11 | 14 | 15 | 17 | 18 | 20 | 21 | 23 | 25 | 27 |
| 90+ | 497 | 8 | 10 | 12 | 13 | 14 | 15 | 16 | 18 | 20 | 22 | 25 |
| Central and Eastern Europe (pooled *n* = 10,759) | | | | | | | | | | | | |
| 50–54 | 685 | 19 | 23 | 25 | 27 | 28 | 29 | 30 | 32 | 33 | 36 | 39 |
| 55–59 | 1,548 | 19 | 21 | 24 | 26 | 28 | 29 | 30 | 32 | 33 | 36 | 38 |
| 60–64 | 1,933 | 17 | 20 | 22 | 25 | 26 | 27 | 29 | 30 | 32 | 35 | 37 |
| 65–69 | 2,205 | 16 | 19 | 22 | 23 | 25 | 26 | 28 | 30 | 31 | 34 | 36 |
| 70–74 | 1,947 | 14 | 16 | 19 | 21 | 23 | 24 | 26 | 27 | 30 | 32 | 35 |
| 75–79 | 1,245 | 12 | 14 | 17 | 19 | 20 | 22 | 24 | 25 | 27 | 30 | 33 |
| 80–84 | 771 | 10 | 12 | 15 | 17 | 18 | 20 | 21 | 23 | 24 | 28 | 30 |
| 85–89 | 327 | 10 | 11 | 13 | 15 | 16 | 17 | 19 | 21 | 23 | 25 | 27 |
| 90+ | 98 | 4 | 8 | 11 | 12 | 13 | 15 | 16 | 18 | 20 | 20 | 24 |
| Northern Europe (pooled *n* = 7,229) | | | | | | | | | | | | |
| 50–54 | 423 | 24 | 25 | 26 | 28 | 30 | 31 | 34 | 35 | 37 | 39 | 41 |
| 55–59 | 828 | 21 | 23 | 26 | 28 | 30 | 30 | 32 | 33 | 35 | 38 | 40 |
| 60–64 | 1,104 | 20 | 22 | 24 | 25 | 27 | 29 | 30 | 32 | 34 | 36 | 38 |
| 65–69 | 1,224 | 18 | 20 | 23 | 25 | 26 | 27 | 28 | 30 | 32 | 34 | 37 |
| 70–74 | 1,218 | 18 | 20 | 22 | 24 | 25 | 26 | 27 | 29 | 30 | 33 | 35 |
| 75–79 | 1,062 | 15 | 17 | 20 | 21 | 23 | 24 | 25 | 27 | 28 | 31 | 33 |
| 80–84 | 811 | 13 | 15 | 17 | 19 | 20 | 21 | 23 | 24 | 26 | 28 | 30 |
| 85–89 | 405 | 10 | 12 | 15 | 17 | 18 | 20 | 21 | 22 | 24 | 26 | 29 |
| 90+ | 154 | 9 | 11 | 12 | 14 | 15 | 16 | 18 | 19 | 20 | 24 | 26 |
| Southern Europe (pooled *n* = 5,306) | | | | | | | | | | | | |
| 50–54 | 247 | 19 | 20 | 22 | 25 | 26 | 27 | 29 | 31 | 33 | 36 | 37 |
| 55–59 | 697 | 18 | 20 | 21 | 23 | 25 | 26 | 28 | 30 | 33 | 36 | 37 |
| 60–64 | 939 | 17 | 21 | 23 | 24 | 25 | 27 | 28 | 30 | 31 | 33 | 36 |
| 65–69 | 978 | 15 | 17 | 20 | 22 | 24 | 25 | 26 | 28 | 30 | 32 | 34 |
| 70–74 | 972 | 13 | 16 | 18 | 20 | 21 | 23 | 24 | 25 | 27 | 30 | 32 |
| 75–79 | 779 | 11 | 14 | 15 | 18 | 19 | 21 | 22 | 24 | 25 | 28 | 31 |
| 80–84 | 431 | 11 | 13 | 15 | 17 | 18 | 20 | 21 | 23 | 25 | 28 | 30 |
| 85–89 | 191 | 7 | 10 | 12 | 14 | 15 | 16 | 18 | 20 | 22 | 25 | 28 |
| 90+ | 72 | 8 | 9 | 10 | 11 | 12 | 14 | 15 | 17 | 18 | 18 | 20 |
| Western Europe (pooled *n* = 9,507) | | | | | | | | | | | | |
| 50–54 | 581 | 20 | 21 | 25 | 27 | 28 | 30 | 31 | 33 | 35 | 38 | 40 |
| 55–59 | 1,255 | 20 | 22 | 25 | 27 | 28 | 29 | 31 | 32 | 34 | 36 | 39 |
| 60–64 | 1,662 | 19 | 21 | 24 | 25 | 27 | 28 | 30 | 31 | 33 | 36 | 38 |
| 65–69 | 1,749 | 18 | 20 | 23 | 24 | 25 | 26 | 28 | 30 | 31 | 34 | 36 |
| 70–74 | 1,643 | 17 | 19 | 21 | 22 | 24 | 25 | 27 | 28 | 30 | 32 | 34 |
| 75–79 | 1,197 | 15 | 17 | 19 | 20 | 22 | 23 | 25 | 26 | 28 | 30 | 31 |
| 80–84 | 858 | 13 | 15 | 17 | 18 | 20 | 21 | 23 | 24 | 25 | 28 | 29 |
| 85–89 | 389 | 10 | 13 | 15 | 16 | 17 | 19 | 20 | 22 | 23 | 26 | 27 |
| 90+ | 173 | 9 | 10 | 12 | 13 | 15 | 16 | 16 | 19 | 21 | 25 | 26 |
| The following classification of countries to European regions was used: Central and Eastern Europe (Bulgaria, Croatia, Czech Republic, Hungary, Poland, Romania, Slovakia, and Slovenia); Northern Europe (Denmark, Estonia, Finland, Latvia, Lithuania, and Sweden); Southern Europe (Cyprus, Greece, Italy, Malta, Portugal, and Spain); and Western Europe (Austria, Belgium, France, Germany, Luxembourg, Netherlands, and Switzerland) | | | | | | | | | | | | |
