# Supplementary material for: Reference values for handgrip strength in Europe: analysis of individual participant data from 27 countries
Source: GeroScience. 2025 Oct 1;48(3):4349–71. doi: 10.1007/s11357-025-01919-9 (PMC13355996; doi:10.1007/s11357-025-01919-9)
Supplement: Supplementary file 2 — (DOCX 22 KB) [file 11357_2025_1919_MOESM2_ESM.docx]

**Electronic Supplementary Material Appendix S2.** Reference values for absolute handgrip strength among men: combined data for testing in standing and sitting positions

| **Age (years)** | ***n*** | **Weighted percentile** **(kg)** | | | | | | | | | | |
| --- | --- | --- | --- | --- | --- | --- | --- | --- | --- | --- | --- | --- |
|  |  | **5^th^** | **10^th^** | **20^th^** | **30^th^** | **40^th^** | **50^th^** | **60^th^** | **70^th^** | **80^th^** | **90^th^** | **95^th^** |
| Europe (pooled *n* = 25,708) | | | | | | | | | | | | |
| 50–54 | 1,078 | 30 | 35 | 39 | 41 | 44 | 46 | 48 | 51 | 54 | 58 | 62 |
| 55–59 | 3,072 | 30 | 34 | 37 | 40 | 42 | 45 | 47 | 50 | 52 | 56 | 59 |
| 60–64 | 4,436 | 27 | 31 | 36 | 40 | 42 | 44 | 46 | 48 | 51 | 55 | 57 |
| 65–69 | 5,116 | 27 | 31 | 35 | 37 | 40 | 42 | 44 | 46 | 49 | 52 | 55 |
| 70–74 | 4,929 | 25 | 28 | 32 | 35 | 37 | 39 | 41 | 44 | 46 | 49 | 52 |
| 75–79 | 3,525 | 22 | 25 | 29 | 31 | 34 | 36 | 38 | 40 | 43 | 46 | 48 |
| 80–84 | 2,215 | 20 | 22 | 27 | 30 | 31 | 33 | 35 | 38 | 40 | 43 | 45 |
| 85–89 | 1,000 | 17 | 20 | 23 | 25 | 28 | 29 | 31 | 33 | 36 | 40 | 43 |
| 90+ | 337 | 12 | 17 | 21 | 23 | 25 | 27 | 29 | 30 | 33 | 36 | 39 |
| Central and Eastern Europe (pooled *n* = 8,182) | | | | | | | | | | | | |
| 50–54 | 312 | 27 | 29 | 36 | 40 | 42 | 45 | 46 | 49 | 52 | 59 | 62 |
| 55–59 | 1,067 | 26 | 31 | 35 | 39 | 42 | 45 | 46 | 48 | 50 | 55 | 58 |
| 60–64 | 1,571 | 25 | 30 | 34 | 37 | 40 | 42 | 45 | 46 | 49 | 53 | 56 |
| 65–69 | 1,789 | 24 | 28 | 33 | 35 | 38 | 40 | 42 | 45 | 47 | 51 | 54 |
| 70–74 | 1,644 | 22 | 26 | 31 | 34 | 35 | 37 | 39 | 41 | 44 | 48 | 50 |
| 75–79 | 962 | 18 | 22 | 28 | 30 | 33 | 35 | 37 | 40 | 42 | 45 | 48 |
| 80–84 | 527 | 15 | 19 | 25 | 28 | 30 | 32 | 34 | 36 | 40 | 43 | 45 |
| 85–89 | 247 | 15 | 19 | 22 | 25 | 27 | 30 | 32 | 35 | 37 | 43 | 45 |
| 90+ | 63 | 12 | 16 | 18 | 20 | 22 | 24 | 26 | 29 | 30 | 37 | 39 |
| Northern Europe (pooled *n* = 5,266) | | | | | | | | | | | | |
| 50–54 | 282 | 32 | 39 | 44 | 46 | 50 | 52 | 53 | 55 | 58 | 62 | 64 |
| 55–59 | 652 | 35 | 39 | 41 | 44 | 46 | 48 | 50 | 53 | 56 | 58 | 63 |
| 60–64 | 885 | 30 | 36 | 40 | 42 | 45 | 47 | 49 | 51 | 54 | 58 | 62 |
| 65–69 | 942 | 28 | 32 | 37 | 40 | 42 | 44 | 46 | 48 | 50 | 54 | 58 |
| 70–74 | 933 | 28 | 32 | 35 | 38 | 40 | 42 | 44 | 46 | 48 | 52 | 54 |
| 75–79 | 743 | 26 | 29 | 32 | 35 | 37 | 39 | 41 | 43 | 45 | 48 | 50 |
| 80–84 | 515 | 23 | 26 | 30 | 31 | 34 | 35 | 37 | 40 | 41 | 45 | 49 |
| 85–89 | 225 | 19 | 22 | 26 | 27 | 29 | 31 | 33 | 35 | 37 | 40 | 44 |
| 90+ | 89 | 15 | 16 | 20 | 23 | 25 | 26 | 28 | 30 | 33 | 38 | 45 |
| Southern Europe (pooled *n* = 4,322) | | | | | | | | | | | | |
| 50–54 | 92 | 28 | 35 | 37 | 40 | 41 | 43 | 45 | 48 | 49 | 54 | 55 |
| 55–59 | 421 | 28 | 32 | 36 | 39 | 41 | 42 | 44 | 46 | 49 | 51 | 55 |
| 60–64 | 669 | 25 | 27 | 34 | 37 | 40 | 42 | 44 | 47 | 49 | 52 | 54 |
| 65–69 | 885 | 25 | 28 | 32 | 34 | 36 | 39 | 41 | 43 | 45 | 49 | 52 |
| 70–74 | 836 | 24 | 26 | 30 | 32 | 35 | 37 | 39 | 40 | 43 | 47 | 50 |
| 75–79 | 709 | 19 | 23 | 26 | 29 | 31 | 33 | 35 | 38 | 40 | 43 | 45 |
| 80–84 | 445 | 18 | 20 | 24 | 27 | 29 | 30 | 32 | 35 | 37 | 40 | 43 |
| 85–89 | 210 | 15 | 17 | 21 | 24 | 26 | 29 | 30 | 32 | 34 | 39 | 42 |
| 90+ | 55 | 9 | 12 | 22 | 23 | 24 | 26 | 27 | 30 | 31 | 34 | 35 |
| Western Europe (pooled *n* = 7,938) | | | | | | | | | | | | |
| 50–54 | 392 | 33 | 35 | 40 | 42 | 45 | 47 | 49 | 53 | 56 | 60 | 62 |
| 55–59 | 932 | 34 | 36 | 40 | 42 | 44 | 46 | 49 | 52 | 54 | 58 | 61 |
| 60–64 | 1,311 | 31 | 35 | 40 | 42 | 45 | 46 | 48 | 50 | 53 | 56 | 59 |
| 65–69 | 1,500 | 30 | 33 | 37 | 40 | 42 | 44 | 46 | 48 | 50 | 54 | 57 |
| 70–74 | 1,516 | 28 | 32 | 35 | 37 | 39 | 41 | 43 | 45 | 48 | 50 | 53 |
| 75–79 | 1,111 | 24 | 27 | 31 | 34 | 35 | 37 | 40 | 42 | 44 | 47 | 49 |
| 80–84 | 728 | 21 | 25 | 29 | 30 | 33 | 35 | 37 | 39 | 41 | 44 | 46 |
| 85–89 | 318 | 19 | 22 | 24 | 26 | 28 | 30 | 31 | 34 | 37 | 40 | 42 |
| 90+ | 130 | 15 | 20 | 22 | 25 | 27 | 28 | 30 | 32 | 34 | 38 | 40 |
| The following classification of countries to regions was used: Central and Eastern Europe (Bulgaria, Croatia, Czech Republic, Hungary, Poland, Romania, Slovakia, and Slovenia); Northern Europe (Denmark, Estonia, Finland, Latvia, Lithuania, and Sweden); Southern Europe (Cyprus, Greece, Italy, Malta, Portugal, and Spain); Western Europe (Austria, Belgium, France, Germany, Luxembourg, Netherlands, and Switzerland) | | | | | | | | | | | | |
