# Supplementary material for: Reference values for handgrip strength in Europe: analysis of individual participant data from 27 countries
Source: GeroScience. 2025 Oct 1;48(3):4349–71. doi: 10.1007/s11357-025-01919-9 (PMC13355996; doi:10.1007/s11357-025-01919-9)
Supplement: Supplementary file 3 — (DOCX 23 KB) [file 11357_2025_1919_MOESM3_ESM.docx]

**Electronic Supplementary Material Appendix** **S3.** Reference values for relative handgrip strength among women: combined data for testing in standing and sitting positions

| **Age (years)** | ***n*** | **Weighted percentile** **(kg/m^2^)** | | | | | | | | | | |
| --- | --- | --- | --- | --- | --- | --- | --- | --- | --- | --- | --- | --- |
|  |  | **5^th^** | **10^th^** | **20^th^** | **30^th^** | **40^th^** | **50^th^** | **60^th^** | **70^th^** | **80^th^** | **90^th^** | **95^th^** |
| Europe (pooled *n* = 32,546) | | | | | | | | | | | | |
| 50–54 | 1,926 | 7.2 | 8.0 | 8.9 | 9.6 | 10.2 | 10.7 | 11.3 | 11.7 | 12.6 | 13.6 | 14.2 |
| 55–59 | 4,309 | 7.3 | 8.0 | 8.8 | 9.5 | 9.9 | 10.5 | 11.1 | 11.6 | 12.3 | 13.3 | 14.0 |
| 60–64 | 5,608 | 6.6 | 7.8 | 8.8 | 9.3 | 9.9 | 10.3 | 10.9 | 11.4 | 12.0 | 13.0 | 13.8 |
| 65–69 | 6,125 | 6.2 | 7.2 | 8.1 | 8.8 | 9.3 | 9.8 | 10.3 | 10.9 | 11.6 | 12.4 | 13.2 |
| 70–74 | 5,749 | 5.8 | 6.6 | 7.6 | 8.2 | 8.8 | 9.2 | 9.8 | 10.4 | 11.0 | 12.0 | 12.7 |
| 75–79 | 4,242 | 4.9 | 5.7 | 6.7 | 7.4 | 8.0 | 8.5 | 9.0 | 9.7 | 10.3 | 11.2 | 12.0 |
| 80–84 | 2,825 | 4.7 | 5.2 | 6.2 | 6.7 | 7.4 | 7.8 | 8.4 | 8.9 | 9.5 | 10.4 | 11.2 |
| 85–89 | 1,284 | 3.7 | 4.3 | 5.3 | 5.9 | 6.5 | 7.1 | 7.7 | 8.3 | 9.0 | 9.9 | 10.7 |
| 90+ | 478 | 3.1 | 3.8 | 4.4 | 4.9 | 5.5 | 5.9 | 6.7 | 7.0 | 7.3 | 8.8 | 9.7 |
| Central and Eastern Europe (pooled *n* = 10,685) | | | | | | | | | | | | |
| 50–54 | 682 | 7.0 | 8.2 | 9.1 | 9.8 | 10.0 | 10.5 | 11.2 | 11.6 | 12.2 | 13.3 | 14.5 |
| 55–59 | 1,540 | 7.1 | 7.8 | 8.9 | 9.7 | 10.3 | 10.8 | 11.1 | 11.7 | 12.4 | 13.3 | 14.3 |
| 60–64 | 1,924 | 6.3 | 7.3 | 8.6 | 9.2 | 9.8 | 10.3 | 10.8 | 11.3 | 12.1 | 13.0 | 13.8 |
| 65–69 | 2,193 | 5.9 | 7.0 | 8.1 | 8.8 | 9.4 | 9.9 | 10.4 | 11.0 | 11.8 | 12.6 | 13.5 |
| 70–74 | 1,938 | 5.2 | 6.2 | 7.3 | 8.0 | 8.8 | 9.2 | 9.9 | 10.4 | 11.1 | 12.1 | 13.0 |
| 75–79 | 1,236 | 4.5 | 5.3 | 6.4 | 7.2 | 7.8 | 8.3 | 9.0 | 9.6 | 10.5 | 11.7 | 12.5 |
| 80–84 | 760 | 4.0 | 4.8 | 5.9 | 6.6 | 7.3 | 7.8 | 8.2 | 8.9 | 9.4 | 10.7 | 11.7 |
| 85–89 | 317 | 4.0 | 4.5 | 5.4 | 5.7 | 6.2 | 6.7 | 7.4 | 8.2 | 8.8 | 9.6 | 10.5 |
| 90+ | 95 | 1.6 | 3.0 | 4.3 | 5.0 | 5.5 | 5.5 | 6.4 | 6.9 | 7.3 | 7.9 | 9.3 |
| Northern Europe (pooled *n* = 7,209) | | | | | | | | | | | | |
| 50–54 | 423 | 8.8 | 9.2 | 10.2 | 10.4 | 10.9 | 11.4 | 12.1 | 12.5 | 13.0 | 13.9 | 14.2 |
| 55–59 | 827 | 7.4 | 8.2 | 9.3 | 10.0 | 10.5 | 10.9 | 11.4 | 11.9 | 12.4 | 13.4 | 14.2 |
| 60–64 | 1,101 | 7.2 | 8.1 | 8.9 | 9.4 | 9.9 | 10.5 | 11.0 | 11.7 | 12.2 | 12.9 | 13.8 |
| 65–69 | 1,223 | 6.5 | 7.5 | 8.4 | 9.0 | 9.6 | 10.0 | 10.4 | 11.0 | 11.6 | 12.4 | 13.4 |
| 70–74 | 1,214 | 6.4 | 7.3 | 8.2 | 8.8 | 9.3 | 9.7 | 10.2 | 10.6 | 11.2 | 12.0 | 12.6 |
| 75–79 | 1,058 | 5.6 | 6.6 | 7.4 | 7.9 | 8.3 | 8.8 | 9.4 | 10.0 | 10.7 | 11.4 | 12.1 |
| 80–84 | 808 | 5.0 | 5.7 | 6.3 | 7.1 | 7.6 | 8.2 | 8.6 | 9.1 | 9.7 | 10.7 | 11.3 |
| 85–89 | 404 | 4.2 | 4.8 | 5.7 | 6.5 | 7.1 | 7.5 | 8.3 | 8.8 | 9.2 | 9.8 | 10.8 |
| 90+ | 151 | 3.6 | 4.2 | 4.8 | 5.2 | 5.6 | 6.6 | 7.0 | 7.7 | 8.1 | 9.0 | 10.4 |
| Southern Europe (pooled *n* = 5,194) | | | | | | | | | | | | |
| 50–54 | 243 | 7.1 | 8.0 | 8.6 | 9.2 | 9.8 | 10.3 | 11.1 | 11.3 | 12.5 | 13.6 | 13.9 |
| 55–59 | 694 | 6.9 | 7.8 | 8.3 | 8.8 | 9.3 | 9.9 | 10.6 | 11.3 | 12.1 | 13.6 | 14.2 |
| 60–64 | 927 | 6.7 | 8.0 | 8.8 | 9.3 | 9.7 | 10.2 | 10.7 | 11.4 | 12.0 | 12.5 | 13.6 |
| 65–69 | 965 | 5.7 | 6.7 | 7.7 | 8.4 | 9.0 | 9.6 | 10.0 | 10.5 | 11.3 | 12.3 | 13.4 |
| 70–74 | 958 | 5.2 | 6.2 | 7.0 | 7.8 | 8.2 | 8.7 | 9.1 | 9.9 | 10.4 | 11.8 | 12.5 |
| 75–79 | 757 | 4.5 | 5.2 | 6.2 | 7.0 | 7.6 | 8.2 | 8.7 | 9.2 | 10.0 | 10.9 | 11.6 |
| 80–84 | 407 | 4.3 | 5.3 | 6.1 | 6.6 | 7.2 | 7.7 | 8.1 | 9.0 | 9.8 | 10.6 | 11.3 |
| 85–89 | 178 | 3.1 | 3.9 | 4.4 | 5.3 | 5.9 | 6.2 | 6.9 | 8.0 | 8.7 | 9.8 | 10.9 |
| 90+ | 65 | 3.1 | 3.5 | 4.0 | 4.4 | 4.7 | 5.7 | 6.1 | 6.7 | 7.0 | 7.3 | 7.8 |
| Western Europe (pooled *n* = 9,458) | | | | | | | | | | | | |
| 50–54 | 578 | 7.1 | 7.7 | 8.9 | 9.6 | 10.3 | 10.8 | 11.4 | 12.0 | 12.8 | 13.8 | 14.3 |
| 55–59 | 1,248 | 7.5 | 8.2 | 9.2 | 9.8 | 10.1 | 10.7 | 11.2 | 11.6 | 12.2 | 13.1 | 13.8 |
| 60–64 | 1,656 | 6.8 | 7.8 | 8.7 | 9.4 | 9.9 | 10.5 | 11.0 | 11.5 | 12.0 | 13.1 | 13.9 |
| 65–69 | 1,744 | 6.8 | 7.5 | 8.4 | 9.0 | 9.4 | 9.9 | 10.5 | 11.0 | 11.6 | 12.5 | 13.1 |
| 70–74 | 1,639 | 6.2 | 7.1 | 7.9 | 8.5 | 9.0 | 9.5 | 10.0 | 10.6 | 11.1 | 12.0 | 12.6 |
| 75–79 | 1,191 | 5.5 | 6.4 | 7.3 | 7.8 | 8.4 | 8.8 | 9.2 | 9.9 | 10.4 | 11.2 | 11.9 |
| 80–84 | 850 | 4.8 | 5.3 | 6.4 | 7.0 | 7.5 | 8.1 | 8.5 | 8.9 | 9.5 | 10.3 | 11.1 |
| 85–89 | 385 | 3.8 | 4.9 | 5.6 | 6.2 | 6.9 | 7.4 | 7.9 | 8.7 | 9.1 | 9.9 | 10.4 |
| 90+ | 167 | 3.3 | 4.2 | 4.7 | 5.3 | 5.6 | 6.3 | 6.8 | 7.2 | 8.1 | 9.6 | 10.0 |
| The following classification of countries to European regions was used: Central and Eastern Europe (Bulgaria, Croatia, Czech Republic, Hungary, Poland, Romania, Slovakia, and Slovenia); Northern Europe (Denmark, Estonia, Finland, Latvia, Lithuania, and Sweden); Southern Europe (Cyprus, Greece, Italy, Malta, Portugal, and Spain); and Western Europe (Austria, Belgium, France, Germany, Luxembourg, Netherlands, and Switzerland) | | | | | | | | | | | | |
