# Supplementary material for: Reference values for handgrip strength in Europe: analysis of individual participant data from 27 countries
Source: GeroScience. 2025 Oct 1;48(3):4349–71. doi: 10.1007/s11357-025-01919-9 (PMC13355996; doi:10.1007/s11357-025-01919-9)
Supplement: Supplementary file 4 — (DOCX 23 KB) [file 11357_2025_1919_MOESM4_ESM.docx]

**Electronic Supplementary Material Appendix** **S4.** Reference values for relative handgrip strength among men: combined data for testing in standing and sitting positions

| **Age (years)** | ***n*** | **Weighted percentile** **(kg/m^2^)** | | | | | | | | | | |
| --- | --- | --- | --- | --- | --- | --- | --- | --- | --- | --- | --- | --- |
|  |  | **5^th^** | **10^th^** | **20^th^** | **30^th^** | **40^th^** | **50^th^** | **60^th^** | **70^th^** | **80^th^** | **90^th^** | **95^th^** |
| Europe (pooled *n* = 25,591) | | | | | | | | | | | | |
| 50–54 | 1,075 | 9.3 | 11.1 | 12.5 | 13.2 | 13.9 | 14.9 | 15.5 | 16.2 | 17.2 | 18.3 | 18.9 |
| 55–59 | 3,063 | 9.7 | 10.6 | 12.1 | 13.0 | 13.7 | 14.4 | 15.0 | 15.7 | 16.7 | 17.7 | 18.7 |
| 60–64 | 4,418 | 8.9 | 10.0 | 11.8 | 12.7 | 13.5 | 14.3 | 15.0 | 15.6 | 16.4 | 17.6 | 18.3 |
| 65–69 | 5,097 | 8.9 | 9.9 | 11.4 | 12.2 | 13.0 | 13.5 | 14.2 | 14.9 | 15.7 | 16.8 | 17.6 |
| 70–74 | 4,904 | 8.2 | 9.3 | 10.6 | 11.6 | 12.3 | 12.9 | 13.6 | 14.2 | 15.0 | 16.1 | 17.2 |
| 75–79 | 3,508 | 7.4 | 8.4 | 9.5 | 10.4 | 11.3 | 11.9 | 12.6 | 13.2 | 14.2 | 15.2 | 15.9 |
| 80–84 | 2,201 | 6.7 | 7.8 | 9.0 | 9.8 | 10.6 | 11.3 | 11.8 | 12.6 | 13.2 | 14.2 | 15.4 |
| 85–89 | 994 | 5.9 | 6.8 | 7.8 | 8.5 | 9.3 | 9.9 | 10.4 | 11.3 | 12.2 | 13.1 | 14.2 |
| 90+ | 331 | 4.5 | 5.9 | 7.1 | 7.9 | 8.4 | 9.3 | 9.8 | 10.7 | 11.1 | 12.1 | 12.9 |
| Central and Eastern Europe (pooled *n* = 8,162) | | | | | | | | | | | | |
| 50–54 | 312 | 8.3 | 8.3 | 11.3 | 12.6 | 13.3 | 14.3 | 15.0 | 15.5 | 16.7 | 18.3 | 19.4 |
| 55–59 | 1,064 | 8.8 | 10.2 | 11.7 | 12.9 | 13.8 | 14.5 | 15.2 | 15.8 | 16.6 | 17.8 | 18.9 |
| 60–64 | 1,567 | 8.4 | 9.9 | 11.3 | 12.2 | 13.0 | 13.7 | 14.5 | 15.2 | 16.0 | 17.3 | 18.2 |
| 65–69 | 1,785 | 8.0 | 9.5 | 11.0 | 11.8 | 12.5 | 13.2 | 13.8 | 14.6 | 15.6 | 16.7 | 17.7 |
| 70–74 | 1,639 | 7.2 | 8.8 | 10.3 | 11.1 | 11.8 | 12.3 | 13.1 | 13.8 | 14.5 | 15.7 | 16.9 |
| 75–79 | 961 | 6.0 | 7.6 | 9.2 | 10.3 | 10.8 | 11.7 | 12.4 | 13.1 | 13.9 | 15.2 | 16.1 |
| 80–84 | 526 | 5.4 | 6.8 | 8.6 | 9.3 | 10.3 | 10.8 | 11.6 | 12.3 | 13.3 | 14.8 | 16.2 |
| 85–89 | 246 | 5.3 | 6.2 | 7.3 | 8.6 | 9.4 | 10.0 | 10.7 | 11.8 | 12.8 | 14.7 | 15.9 |
| 90+ | 62 | 4.7 | 5.7 | 6.8 | 6.9 | 7.8 | 8.5 | 8.8 | 10.5 | 10.7 | 11.5 | 12.5 |
| Northern Europe (pooled *n* = 5,257) | | | | | | | | | | | | |
| 50–54 | 281 | 9.9 | 12.0 | 13.3 | 14.2 | 15.1 | 15.6 | 16.4 | 17.3 | 18.3 | 19.0 | 20.5 |
| 55–59 | 650 | 10.8 | 11.6 | 12.4 | 13.6 | 14.3 | 14.9 | 15.6 | 16.5 | 17.1 | 18.2 | 19.2 |
| 60–64 | 883 | 9.3 | 11.1 | 12.4 | 13.1 | 13.9 | 14.6 | 15.4 | 16.1 | 17.1 | 18.0 | 18.9 |
| 65–69 | 942 | 9.1 | 10.5 | 11.6 | 12.5 | 13.2 | 13.6 | 14.2 | 15.0 | 15.8 | 16.9 | 18.0 |
| 70–74 | 932 | 8.7 | 10.2 | 11.4 | 12.2 | 12.9 | 13.3 | 13.9 | 14.5 | 15.2 | 16.3 | 17.3 |
| 75–79 | 742 | 8.6 | 9.4 | 10.3 | 11.2 | 11.8 | 12.3 | 13.0 | 13.6 | 14.2 | 15.2 | 16.0 |
| 80–84 | 513 | 7.5 | 8.5 | 9.4 | 10.3 | 10.8 | 11.3 | 11.9 | 12.5 | 13.2 | 14.4 | 15.5 |
| 85–89 | 225 | 6.3 | 7.3 | 8.5 | 9.0 | 9.4 | 9.8 | 10.3 | 11.2 | 12.1 | 13.0 | 13.9 |
| 90+ | 89 | 5.0 | 5.4 | 6.7 | 7.6 | 8.4 | 9.1 | 9.5 | 10.1 | 10.9 | 12.4 | 14.5 |
| Southern Europe (pooled *n* = 4,266) | | | | | | | | | | | | |
| 50–54 | 91 | 8.2 | 11.7 | 12.1 | 13.1 | 13.4 | 14.0 | 15.4 | 15.5 | 16.0 | 16.5 | 18.0 |
| 55–59 | 418 | 9.7 | 9.9 | 11.7 | 12.2 | 13.0 | 13.9 | 14.5 | 14.9 | 15.6 | 17.3 | 18.0 |
| 60–64 | 664 | 8.7 | 8.9 | 11.1 | 12.4 | 12.8 | 13.6 | 14.5 | 15.5 | 16.2 | 17.5 | 18.0 |
| 65–69 | 877 | 8.2 | 9.3 | 10.4 | 11.4 | 12.3 | 13.0 | 13.6 | 14.4 | 15.0 | 16.3 | 17.3 |
| 70–74 | 822 | 8.0 | 8.7 | 9.8 | 10.8 | 11.8 | 12.4 | 13.3 | 13.8 | 14.7 | 16.0 | 17.3 |
| 75–79 | 700 | 6.6 | 8.0 | 8.9 | 9.8 | 10.7 | 11.6 | 12.2 | 12.9 | 13.6 | 14.9 | 15.6 |
| 80–84 | 435 | 6.2 | 7.1 | 8.3 | 9.2 | 10.0 | 10.7 | 11.5 | 12.2 | 13.0 | 13.9 | 14.8 |
| 85–89 | 206 | 5.2 | 6.1 | 7.4 | 8.3 | 9.0 | 9.8 | 10.4 | 11.2 | 12.0 | 13.1 | 14.5 |
| 90+ | 53 | 3.2 | 4.9 | 7.5 | 7.9 | 8.5 | 9.6 | 9.7 | 10.5 | 10.9 | 11.6 | 12.9 |
| Western Europe (pooled *n* = 7,906) | | | | | | | | | | | | |
| 50–54 | 391 | 9.9 | 11.4 | 12.6 | 13.3 | 14.4 | 15.1 | 15.7 | 16.4 | 17.4 | 18.3 | 18.9 |
| 55–59 | 931 | 9.8 | 11.0 | 12.5 | 13.3 | 13.9 | 14.8 | 15.5 | 16.3 | 17.0 | 18.0 | 18.8 |
| 60–64 | 1,304 | 9.8 | 11.2 | 12.6 | 13.4 | 14.2 | 14.9 | 15.4 | 15.9 | 16.7 | 17.6 | 18.6 |
| 65–69 | 1,493 | 9.6 | 10.6 | 11.9 | 12.6 | 13.3 | 13.8 | 14.5 | 15.1 | 16.0 | 17.1 | 18.1 |
| 70–74 | 1,511 | 8.8 | 10.1 | 11.2 | 12.0 | 12.7 | 13.3 | 13.9 | 14.5 | 15.2 | 16.3 | 17.4 |
| 75–79 | 1,105 | 7.9 | 8.8 | 9.9 | 10.8 | 11.7 | 12.1 | 12.8 | 13.6 | 14.5 | 15.4 | 16.1 |
| 80–84 | 727 | 7.6 | 8.4 | 9.5 | 10.2 | 10.9 | 11.4 | 12.2 | 12.8 | 13.5 | 14.7 | 15.6 |
| 85–89 | 317 | 6.5 | 7.1 | 7.9 | 8.8 | 9.4 | 9.9 | 10.4 | 11.4 | 12.1 | 13.1 | 13.8 |
| 90+ | 127 | 5.2 | 6.2 | 7.4 | 8.1 | 8.9 | 9.6 | 10.3 | 10.9 | 11.6 | 12.8 | 14.3 |
| The following classification of countries to European regions was used: Central and Eastern Europe (Bulgaria, Croatia, Czech Republic, Hungary, Poland, Romania, Slovakia, and Slovenia); Northern Europe (Denmark, Estonia, Finland, Latvia, Lithuania, and Sweden); Southern Europe (Cyprus, Greece, Italy, Malta, Portugal, and Spain); and Western Europe (Austria, Belgium, France, Germany, Luxembourg, Netherlands, and Switzerland) | | | | | | | | | | | | |
